# The complete mitochondrial genome of *Mesogobio lachneri* (Cypriniformes: Gobionidae) from Northeast Asia

**DOI:** 10.1080/23802359.2022.2131370

**Published:** 2022-10-19

**Authors:** Wei Tian, Xiaomin Ni, Cuizhang Fu

**Affiliations:** School of Life Sciences, Ministry of Education Key Laboratory for Biodiversity Science and Ecological Engineering, Coastal Ecosystems Research Station of the Yangtze River Estuary, Institute of Biodiversity Science and Institute of Eco-Chongming, Fudan University, Shanghai, China

**Keywords:** Cypriniformes, Gobionidae, *Mesogobio*, *Gobio*, Yalu River

## Abstract

Although *Mesogobio lachneri* is the type species of the genus *Mesogobio*, its systematic position and status have remained unresolved to date. In this study, for the first time, we report the complete mitochondrial genome of *M. lachneri* using Sanger sequencing. It is a circular genome with a length of 16,602 bp, comprising 22 tRNAs, 13 protein-coding genes (PCGs), two rRNAs, and one non-coding control region. Our phylogenetic analysis reveals that *M. lachneri* is the close relative of the genus *Gobio*, indicating that *Mesogobio* may be a valid genus.

## 1. Introduction

**Figure 1.**
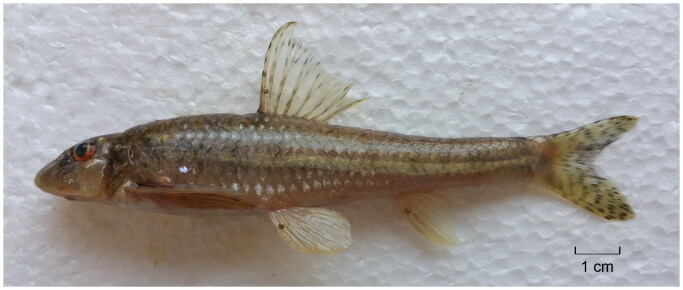
Female *Mesogobio lachneri*. The specimen from the Linjiang City, Jilin Province, China. The photograph by Cuizhang Fu on 22 September 2017.

The freshwater fish genus *Mesogobio* is placed into the order Cypriniformes, the family Gobionidae, and the sub-family Gobioninae (Tan and Armbruster 2018). The genus comprises two species *Mesogobio lachneri* Bănărescu & Nalbant 1973 and *Mesogobio tumenensis* Chang 1980 which are known to be distributed in Northeast Asia (Xie 2007). The results of two prior studies on the molecular phylogeny of the Gobioninae have indicated that *M. tumensis* should be moved into the *Gobio*, as *G. tumensis* (Yang et al. 2006; Tang et al. 2011). However, the type species (*M. lachneri*) of the genus *Mesogobio* has lacked molecular data until now. Hence, the systematic position and status of *M. lachneri* have remained unresolved. *M. lachneri* is a small benthic and rheophilic species with the body length less than 14 cm (Figure 1). It prefers pebbly or sandy substrate environments, and is endemic to the Yalu River (the boundary river between North Korea and China) drainage (Yue 1998; Xie 2007). *M. lachneri* differs from other gobionid species, in having the following six morphological characters: a naked breast, absence of sub-lobes on the lower lip, absence of mental barbels, two rows of teeth, lips with developed papillae, and scale rows above lateral lines 5.5 (Yue 1998).

In the present study, the complete mitochondrial genome of *M. lachneri* is obtained for the first time, which may be used to clarify the phylogenetic position of the genus *Mesogobio* within the family Gobionidae in the future. The mitogenome is also useful as a reference sequence for molecular species identification, as well as for further research on the molecular evolution of the family Gobionidae.

## 2. Materials

*M. lachneri* was collected from the Yalu River in the Linjiang City, Jilin Province, China (41.8087°N, 126.9211°E), and deposited at the Zoological Museum of Fudan University (Cuizhang Fu, czfu@fudan.edu.cn) under the voucher number FDZM-MeLLJ 20170922-01.

## 3. Methods

We extracted genomic DNA from muscle tissues following a high-salt protocol (Miller et al. 1988). The mitochondrial genome fragments were amplified using 13 primer pairs following a protocol previously described in our lab (Chai and Fu 2020; PCR gel image in Figure S1 of Appendix I). For the present study, we designed the forward primer of Primer9 (MeLND4F: 5′-TAGCCAGCCAAAAYCACAT-3′), as well as the reverse primers of Primer8 (MeLND4R: 5′-TAAAATCTGRTGGGCCGG-3′) and Primer10 (MeLND5R: 5′-AAACGRCTTGCCTGRGGAAG-3′). The remaining primers were adopted from Chai and Fu (2020) (Table S1 in Appendix I). The PCR products were sequenced by the Sanger method using the ABI 3730 platform (Applied Biosystems, Foster City, USA; raw sequencing results in Appendix II).

**Table 1.** Characteristics of the mitochondrial genome of *M. lachneri.*

Element	From	To	Length (bp)	Start codon	Stop codon
*tRNA^Phe^*	1	69	69		
*12S rRNA*	70	1028	959		
*tRNA^Val^*	1029	1100	72		
*16S rRNA*	1101	2791	1691		
*tRNA^Leu^*	2792	2867	76		
*ND1*	2869	3843	975	ATG	TAG
*tRNA^Ile^*	3848	3919	72		
*tRNA^Gln^*	3918	3988	71		
*tRNA^Met^*	3990	4058	69		
*ND2*	4059	5103	1045	ATG	T-
*tRNA^Trp^*	5104	5173	70		
*tRNA^Ala^*	5177	5245	69		
*tRNA^Asn^*	5247	5319	73		
*tRNA^Cys^*	5350	5417	68		
*tRNA^Tyr^*	5419	5489	71		
*COI*	5491	7041	1551	GTG	TAA
*tRNA^Ser^*	7042	7112	71		
*tRNA^Asp^*	7116	7187	72		
*COII*	7201	7891	691	ATG	T-
*tRNA^Lys^*	7892	7967	76		
*ATPase8*	7969	8133	165	ATG	TAA
*ATPase6*	8127	8810	684	ATG	TAA
*COIII*	8810	9593	784	ATG	T-
*tRNA^Gly^*	9594	9664	71		
*ND3*	9665	10,014	350	ATG	TA-
*tRNA^Arg^*	10,015	10,084	70		
*ND4L*	10,085	10,381	297	ATG	TAA
*ND4*	10,375	11,756	1382	ATG	TA-
*tRNA^His^*	11,757	11,825	69		
*tRNA^Ser^*	11,826	11,894	69		
*tRNA^Leu^*	11,896	11,968	73		
*ND5*	11,969	13,804	1836	ATG	TAG
*ND6*	13,801	14,322	522	ATG	TAG
*tRNA^Glu^*	14,323	14,391	69		
*Cytb*	14,396	15,536	1141	ATG	T-
*tRNA^Thr^*	15,537	15,608	72		
*tRNA^Pro^*	15,608	15,677	70		
*D-loop*	15,678	16,602	925		

The contiguous and overlapping segments of *M. lachneri* were assembled with default parameters, using Sequencher 5.4 (Gene Codes, Ann Arbor, MI) on the basis of the reference genome of *Gobio macrocephalu*s (MT632636; Yi and Fu 2020). MitoAnnotator was selected as the method for genome annotation (http://mitofish.aori.u-tokyo.ac.jp/annotation/input.html; Iwasaki et al. 2013). The CGView Server was used to generate the genome map (Grant and Stothard 2008).

Tang et al. (2011) have suggested that the genus *Gobio* is a close relative of the *Mesogobio*. Due to the fact that no other mitochondrial genome of fish belonging to the genus *Mesogobio* is available in GenBank, the mitochondrial genomes of five *Gobio* species (*G. gobio*, AB239596, Saitoh et al. 2006; *G. acutipinnatus*, MT632635, Yi and Fu 2020; *G. macrocephalus*, MT632636, Yi and Fu 2020; *G. cynocephalus*, KU314700, Li et al. 2018; and *G. coriparoides*, MN864250, Ge et al. 2020) and one *Romanogobio* species (*R. ciscaucasicus*, AP011259, Iwasaki et al. 2013) were downloaded from GenBank. The 13 protein-coding genes (PCGs) were used in the phylogenetic analyses, and *R. ciscaucasicus* was chosen as the outgroup taxon (Tang et al. 2011). For phylogenetic analyses, the data were partitioned by gene. The maximum-likelihood analysis was conducted using 1000 ultra-fast bootstrap replicates, and the best nucleotide substitution models were selected using the ‘-MF-mtree’ module- implemented in IQ-TREE v2.1.3 (Minh et al. 2020). Bayesian’s analysis was performed using two replicates of 50 million iterations, and the best models were automatically searched using the bModelTest module, implemented in BEAST v2.6.7 (Bouckaert and Drummond 2017).

## 4. Results

**Figure 2.**
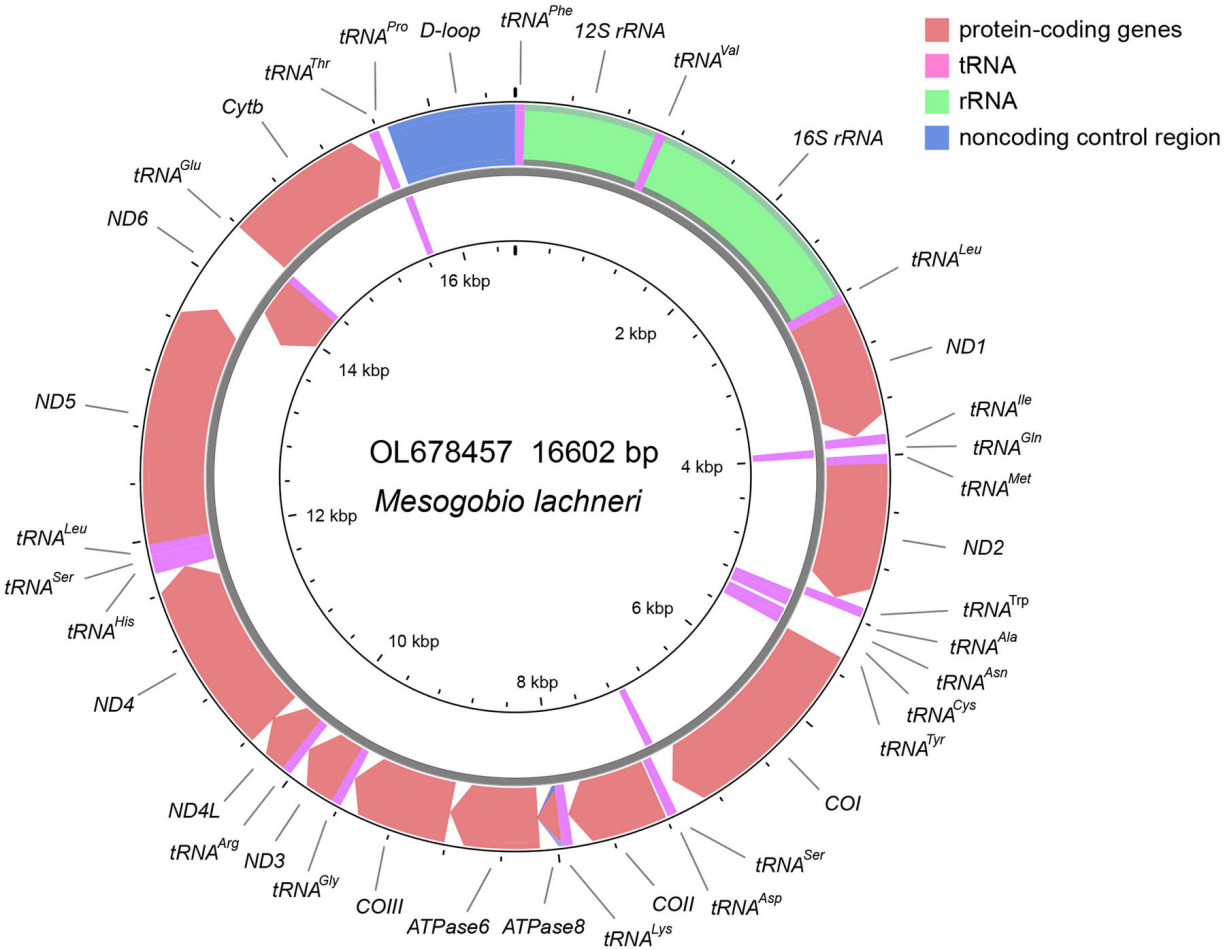
Mitochondrial genome map of *M. lachneri.*

**Figure 3.**
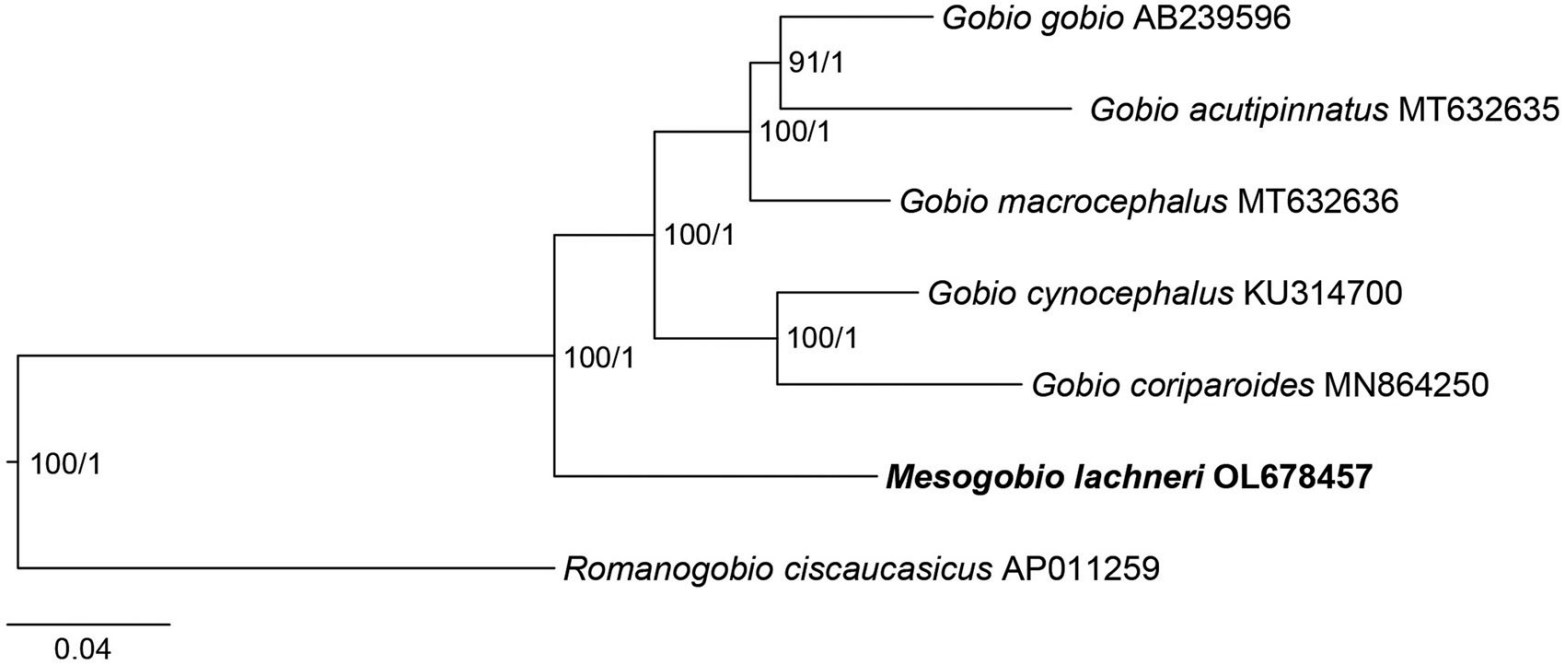
Phylogenetic reconstruction of *M. lachneri* and its close relatives based on 13 mitochondrial protein-coding genes. Numbers near the nodes indicate bootstrap support and posterior probability.

The mitochondrial genome of *M. lachneri* (OL678457) assembled from PCR products, presented as a circular structure with length of 16,602 bp, including a total of 37 genes (22 tRNA genes, 13 PCGs, and two rRNA genes) and one control region (Figure 2). The overall base composition was composed of 30.1% A, 26.4% T, 17.1% G, and 26.5% C. For the 13 PCGs, two types of starting codons (ATG and GTG), and four kinds of stop codons (TAA, TAG, TA-, and T-) were observed (Table 1). Same patterns for codon use, are common in published mitochondrial genomes of the subfamily Gobioninae (Tong and Fu 2019; Zhang and Fu 2019; Fu and Fu 2020; Yi and Fu 2020). Reconstructed phylogenetic relationships between mitogenomes show that *M. lachneri* is the close relative of the genus *Gobio* with strong bootstrap support (100%, Figure 3).

## 5. Discussion and conclusions

The systematic position and status of the genus *Mesogobio* have remained unresolved to date, due to a lack of molecular data for the type species *M. lachneri* (Tang et al. 2011). In the present study, we determine the complete mitochondrial genome of *M. lachneri* for the first time. Our phylogenetic analysis reveals that *M. lachneri* has a close relationship with the genus *Gobio*, indicating that the genus *Mesogobio* may be a valid genus. The provided mitogenome of *M. lachneri* is expected to be useful for molecular species delimitation and for assessment of the molecular evolution of the family Gobionidae in the future.

## Supplementary Material

Supplemental Material

Supplemental Material

## Data Availability

The new genome in this study is openly available in GenBank of NCBI at https://www.ncbi.nlm.nih.gov/nuccore/OL678457. Raw Sanger sequencing data are provided in supplementary material.
