# Supplementary material for: The complete mitochondrial genome of Mesogobio lachneri (Cypriniformes: Gobionidae) from Northeast Asia
Source: Mitochondrial DNA B Resour. 2022 Oct 19;7(10):1810–3. doi: 10.1080/23802359.2022.2131370 (PMC9586598; doi:10.1080/23802359.2022.2131370)

**Supplementary Material—Appendix II**

**Appendix II Raw Sanger sequencing results of fragment 1–13 (primer pair 1–13 used for** **amplification of fragments provided in Table S1)**

1. Fragment 1 was sequenced using primer pair 1 as follow:

Fragment 1-anterior sequence (forward sequencing)


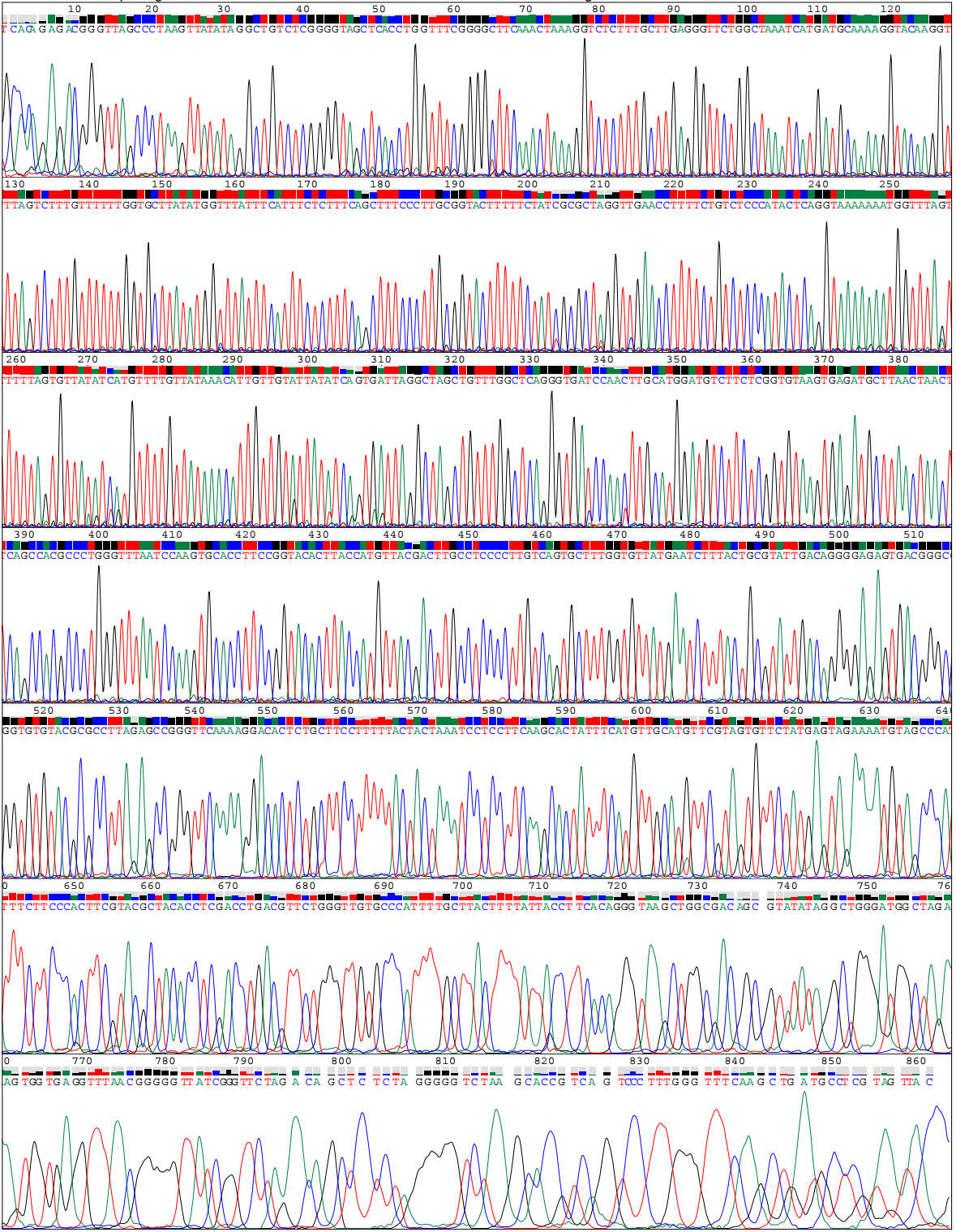


Fragment 1-posterior sequence (reverse sequencing)


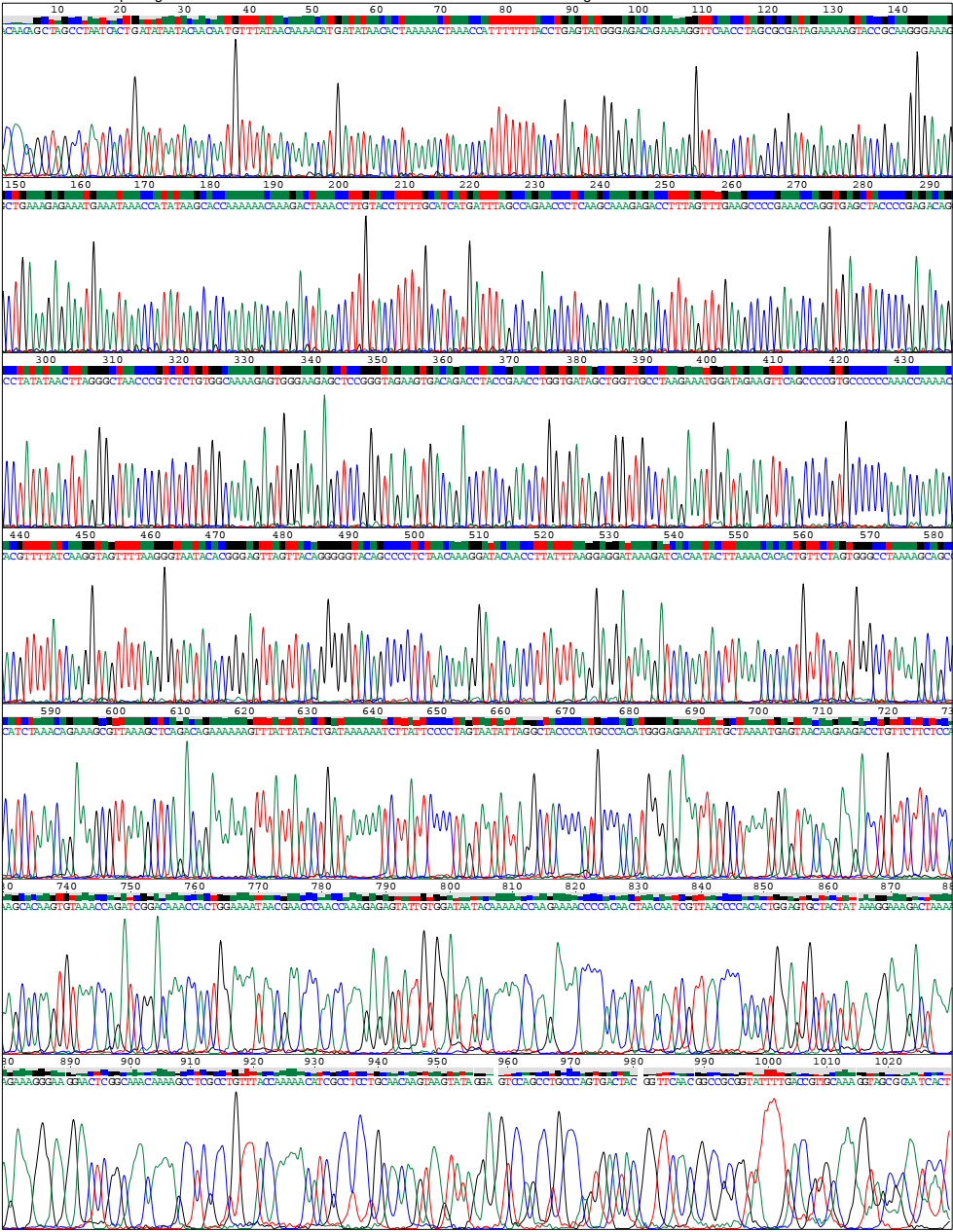


1. Fragment 2-anterior sequence was sequenced using the forward primer of primer pair 2 and Fragment 2-posterior sequence was sequenced using the intermediate primer MeL16SF (5'-AGATCGGACAARCCACTGGA-3') as follow:

Fragment 2-anterior sequence (forward sequencing)


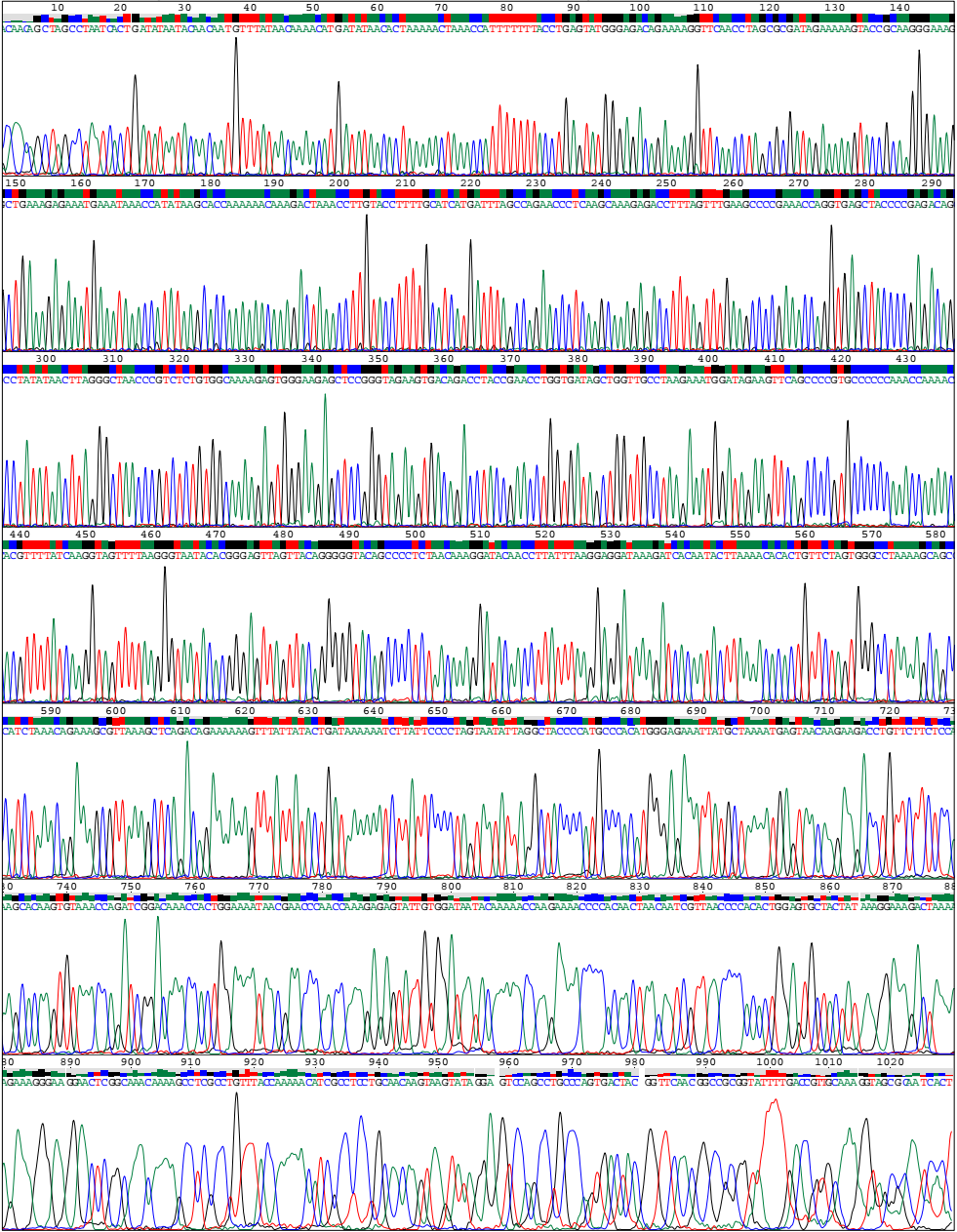


Fragment 2-posterior sequence (forward sequencing)


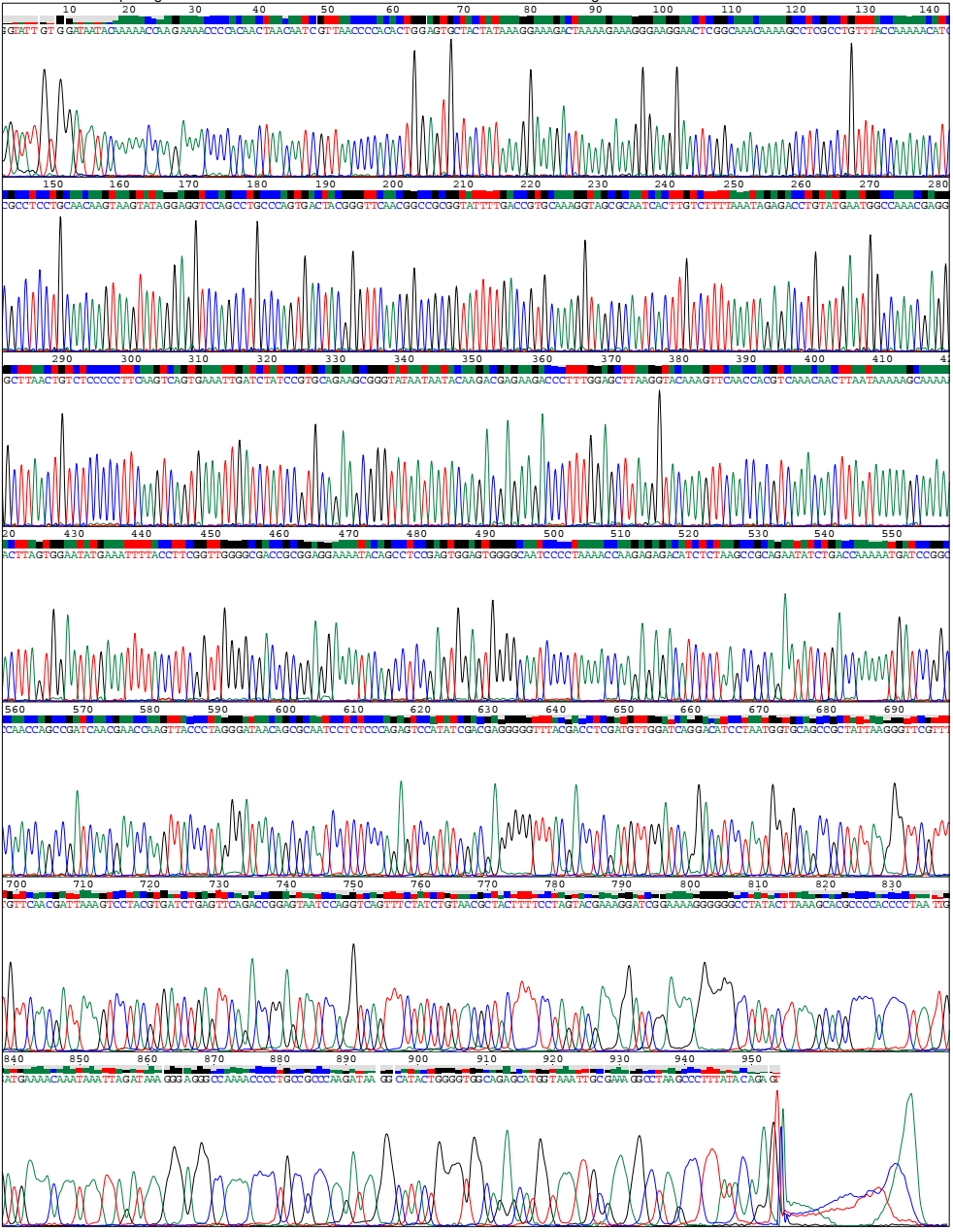


1. Fragment 3 was sequenced using primer pair 3 as follow:

Fragment 3-anterior sequence (forward sequencing)


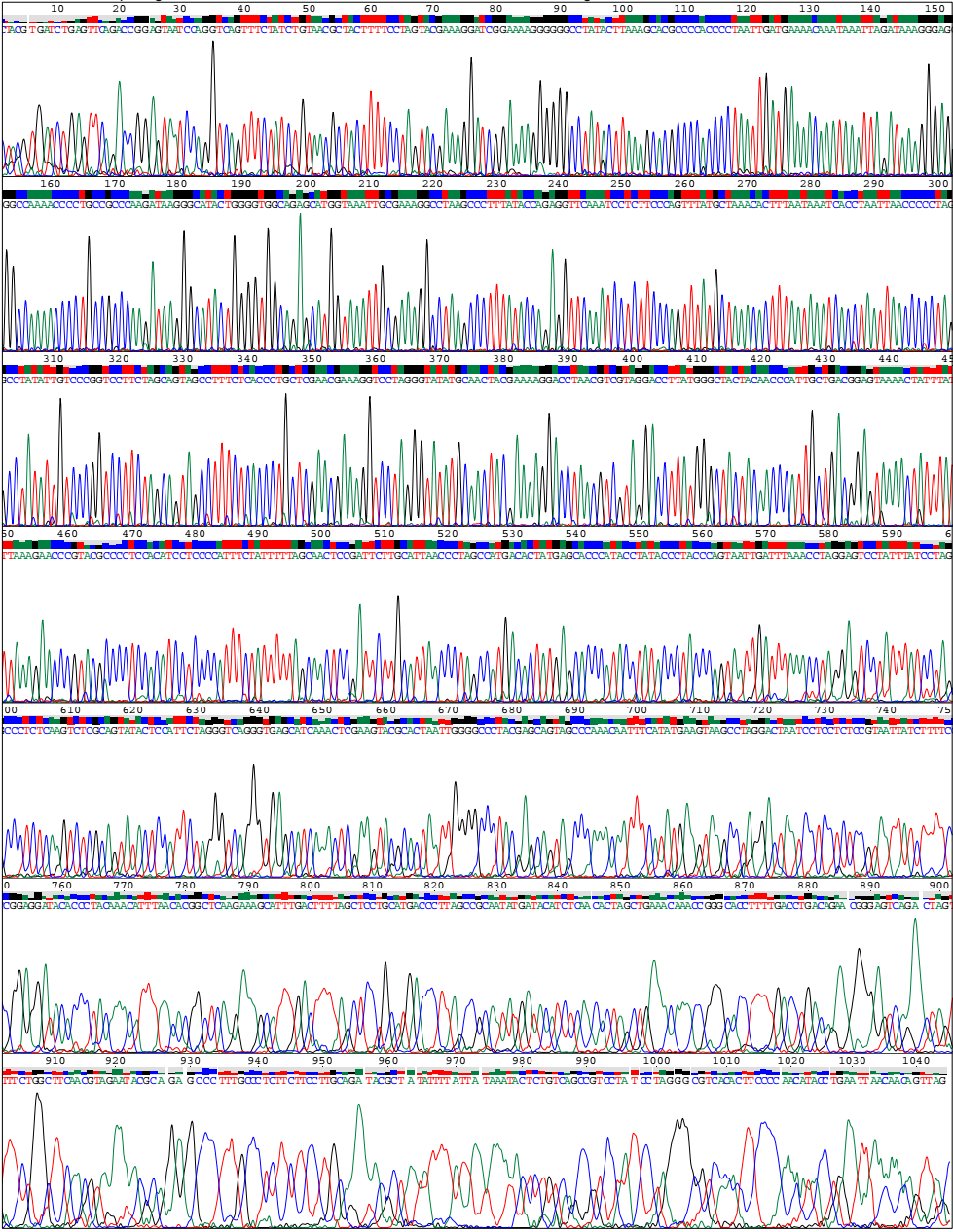


Fragment 3-posterior sequence (reverse sequencing)


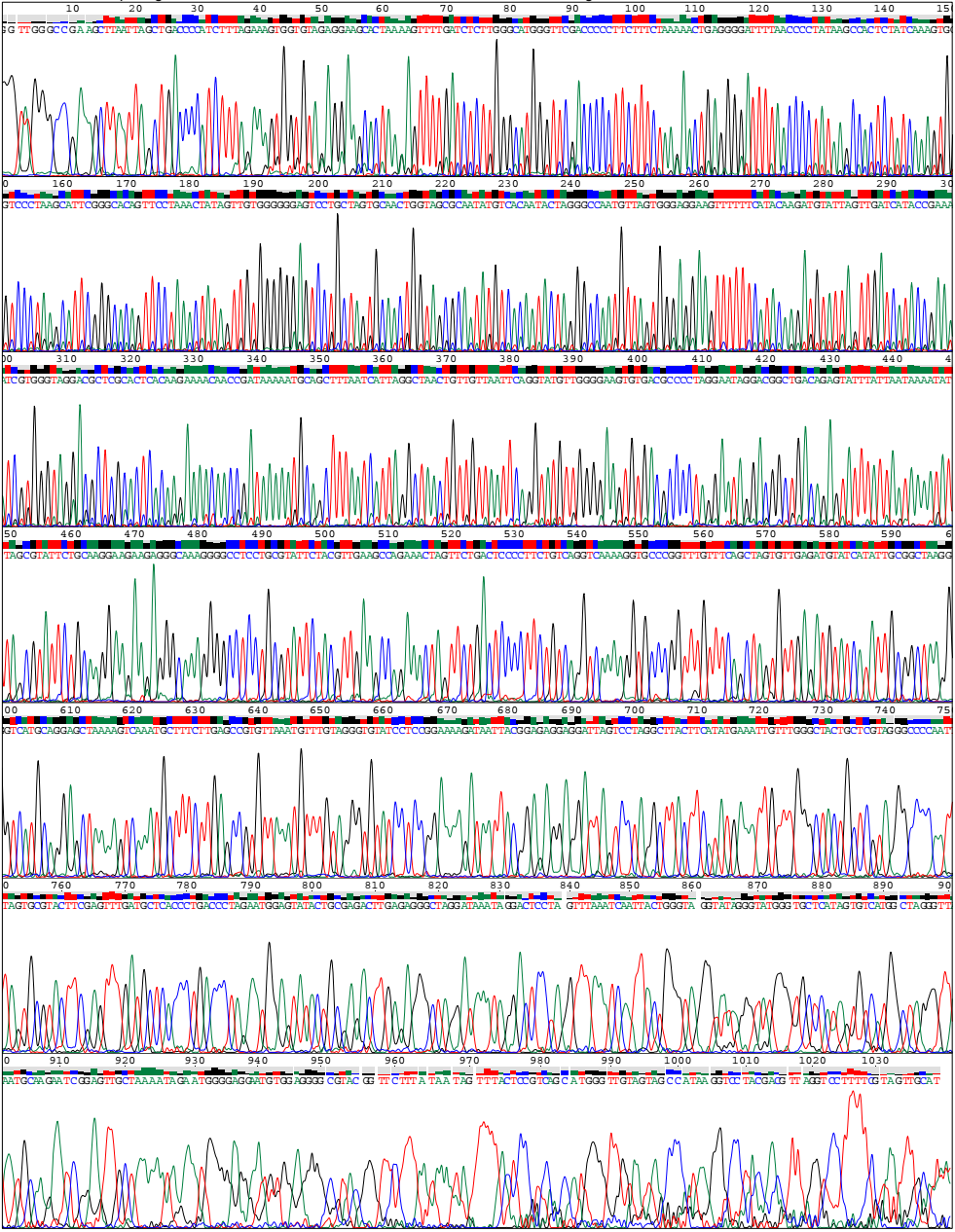


1. Fragment 4 was sequenced using primer pair 4 as follow:

Fragment 4-anterior sequence (forward sequencing)


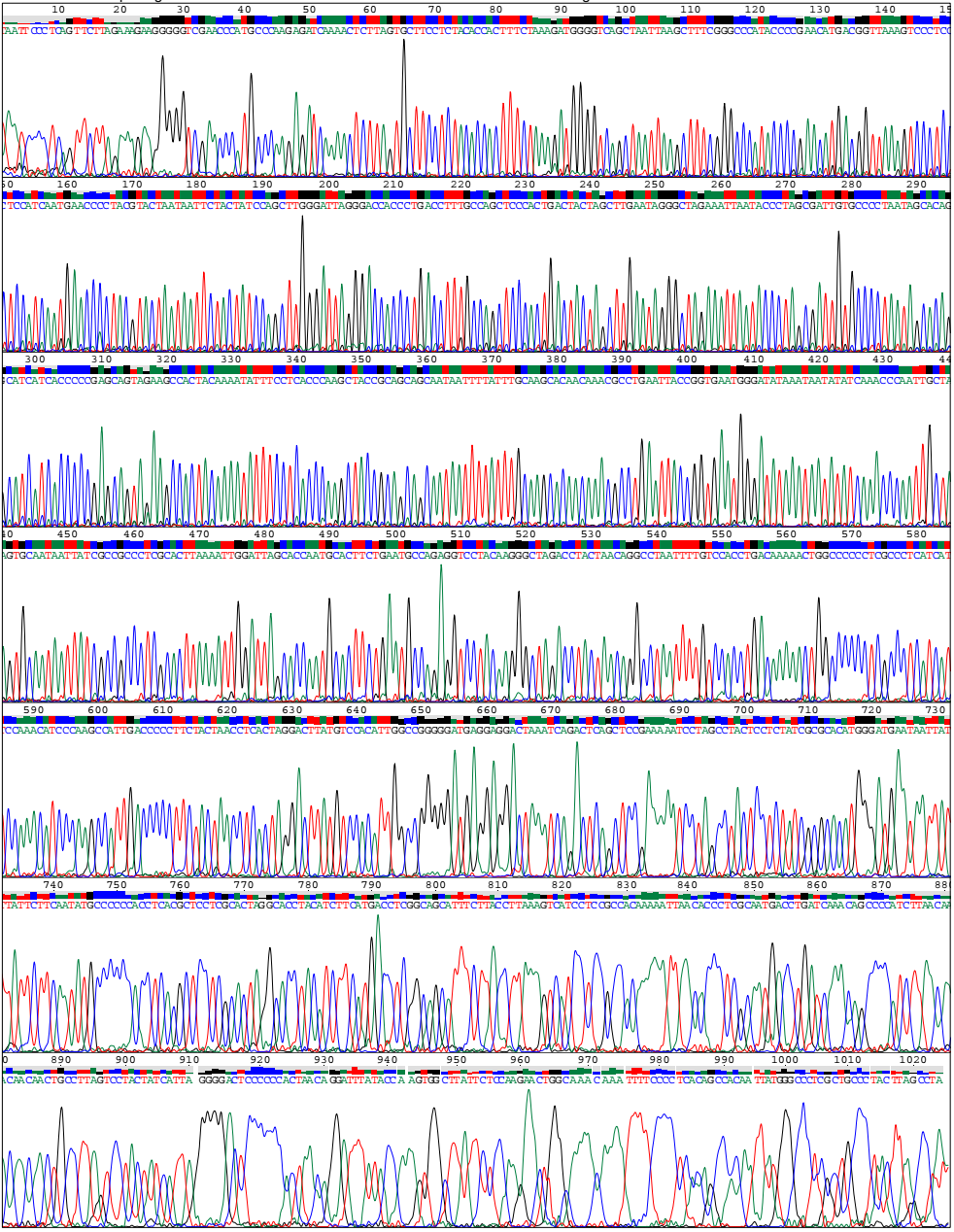


Fragment 4-posterior sequence (reverse sequencing)


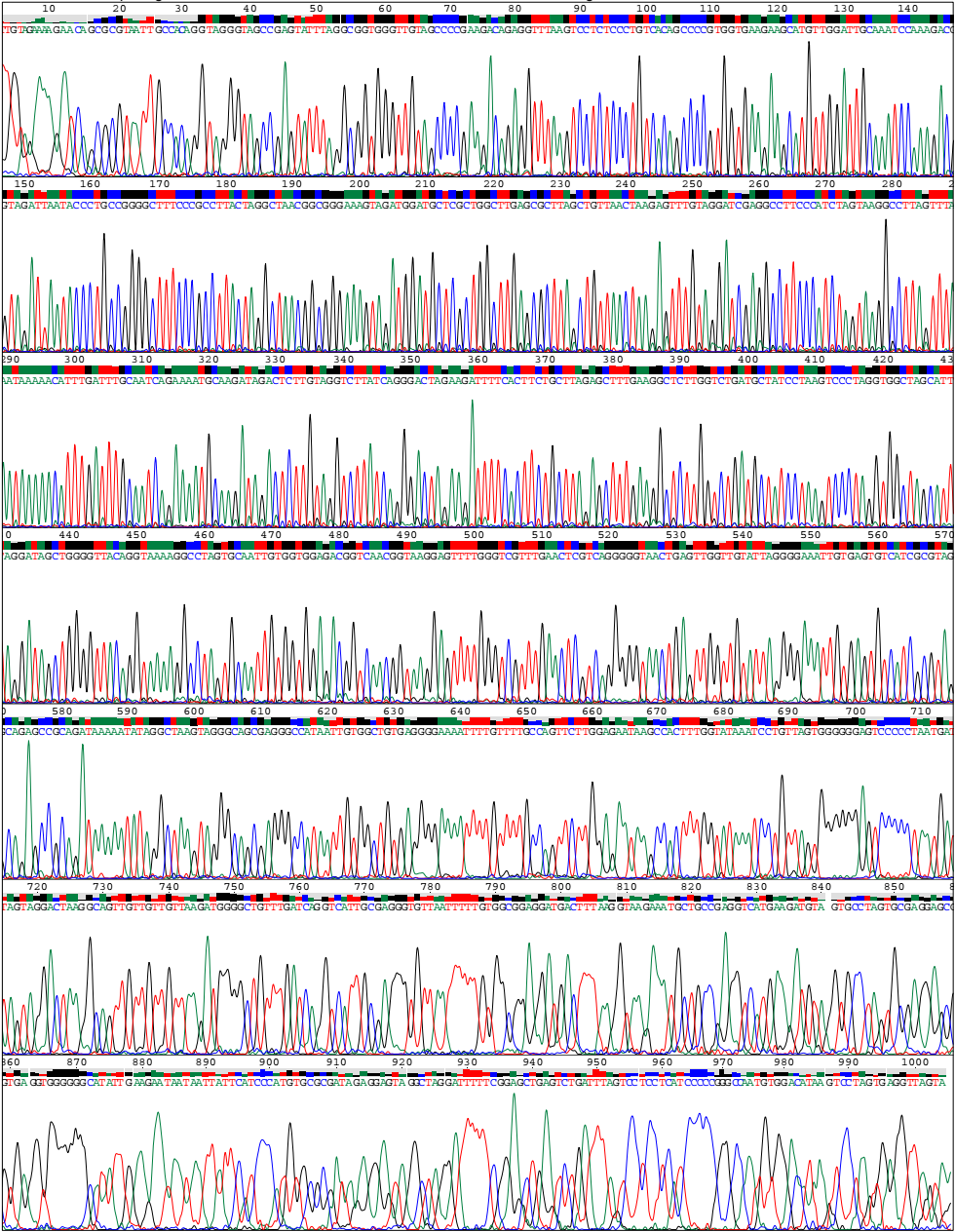


1. Fragment 5 was sequenced using primer pair 5 as follow:

Fragment 5-anterior sequence (forward sequencing)


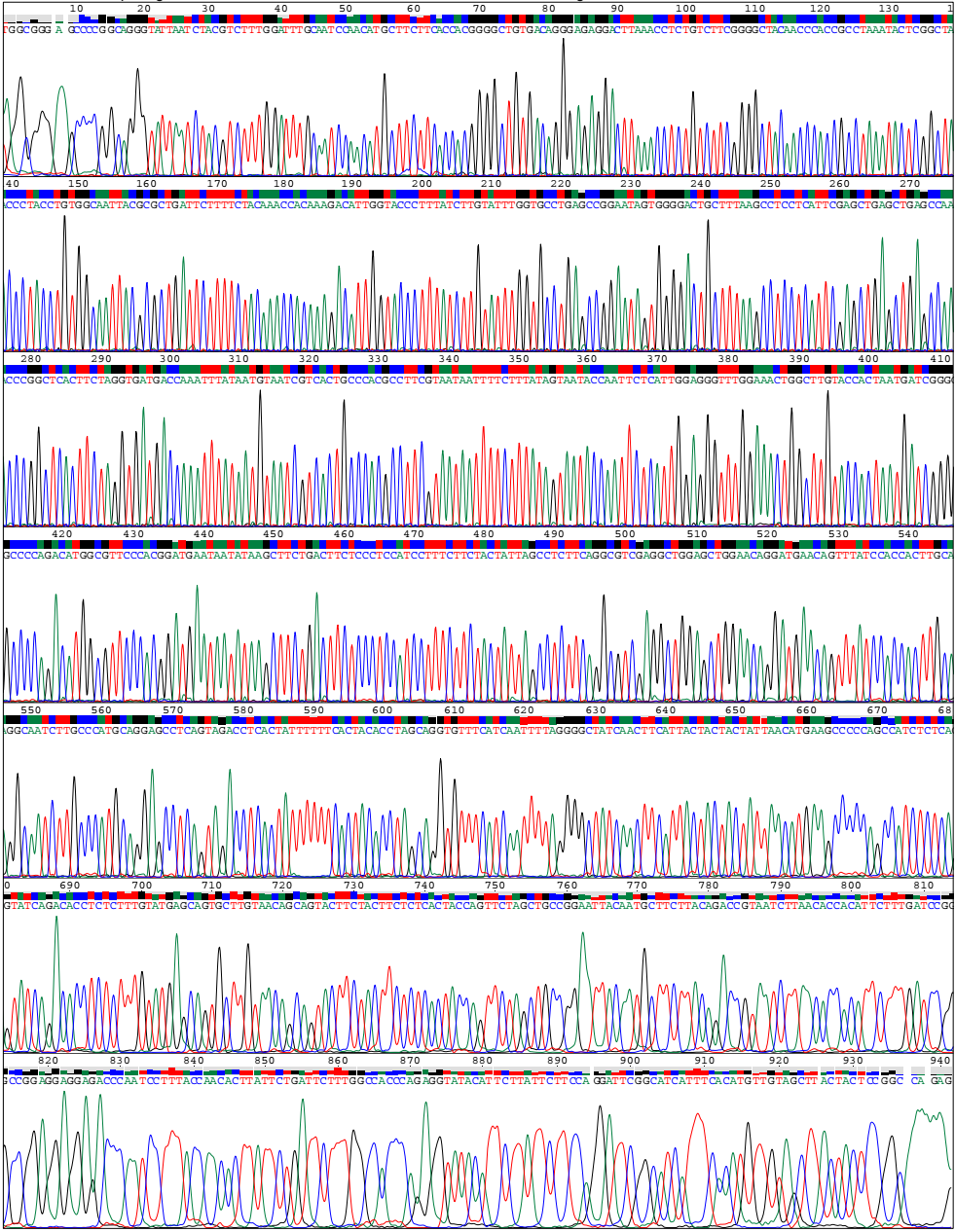


Fragment 5-posterior sequence (reverse sequencing)


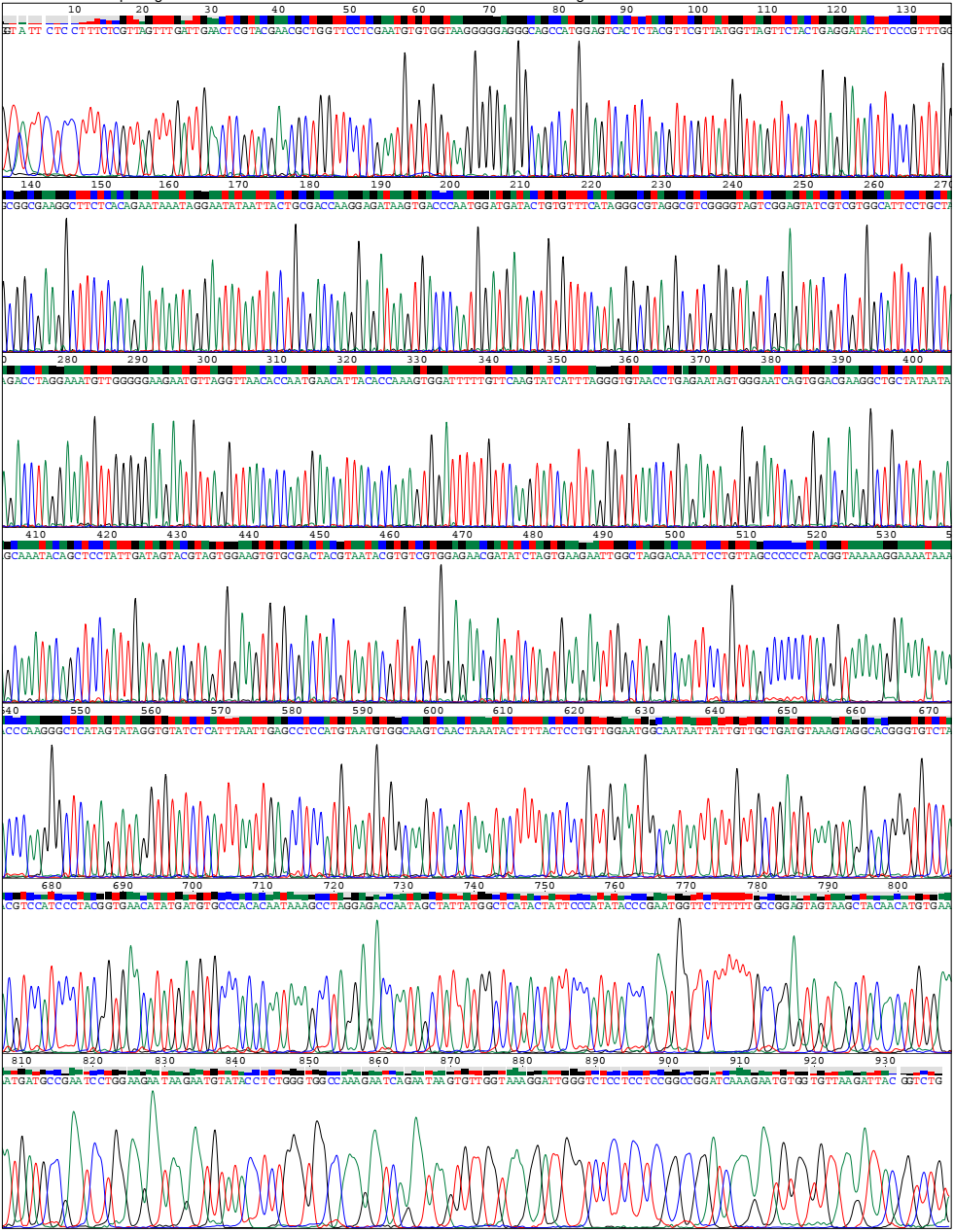


1. Fragment 6-anterior sequence was sequenced using the intermediate primer MeLATP8R (5'-ACTAGAGGTGGTCGGKAGTCA-3') and Fragment 6-posterior sequence was sequenced using the reverse primer of primer pair 6 as follow:

Fragment 6-anterior sequence (reverse sequencing)


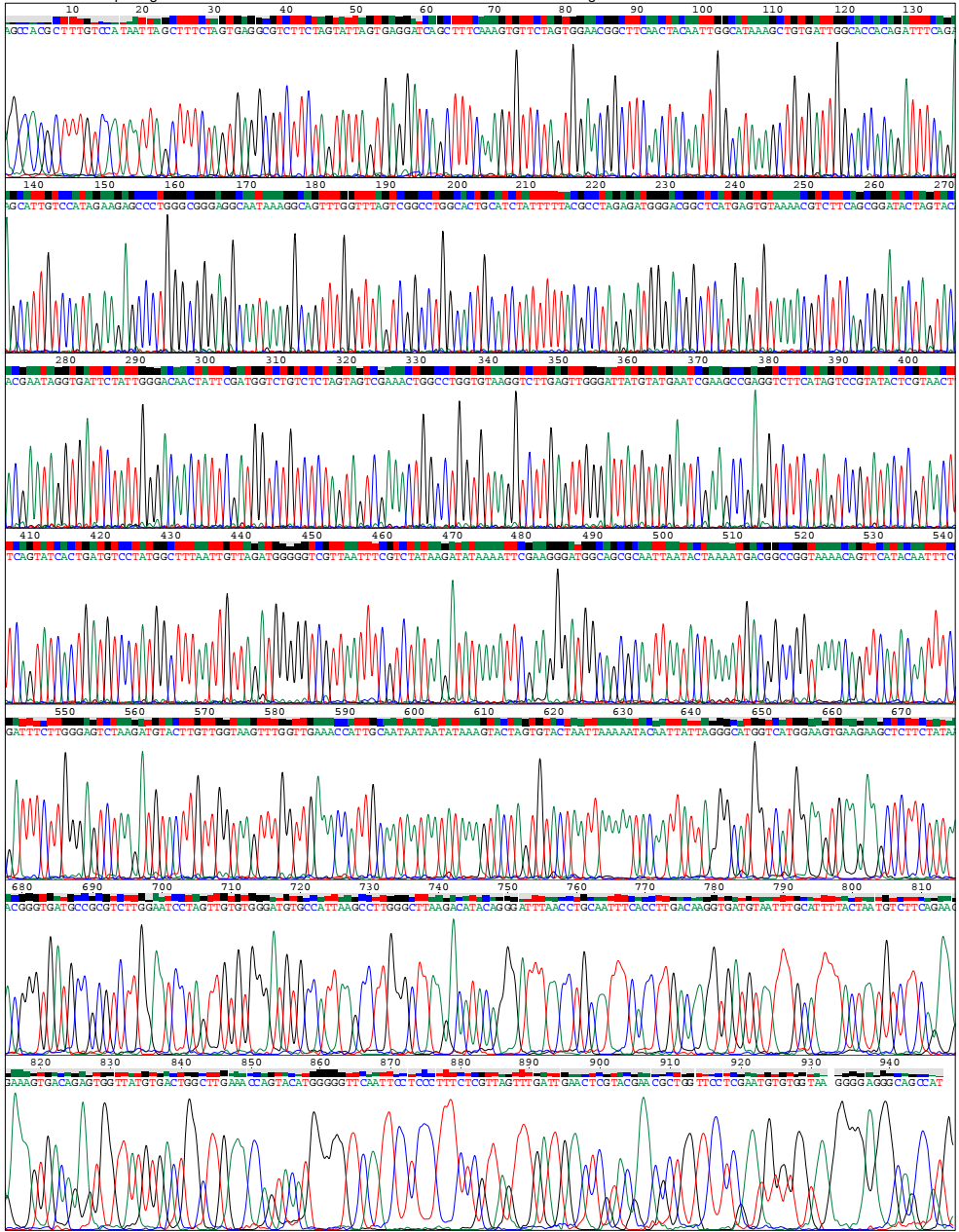


Fragment 6-posterior sequence (reverse sequencing)


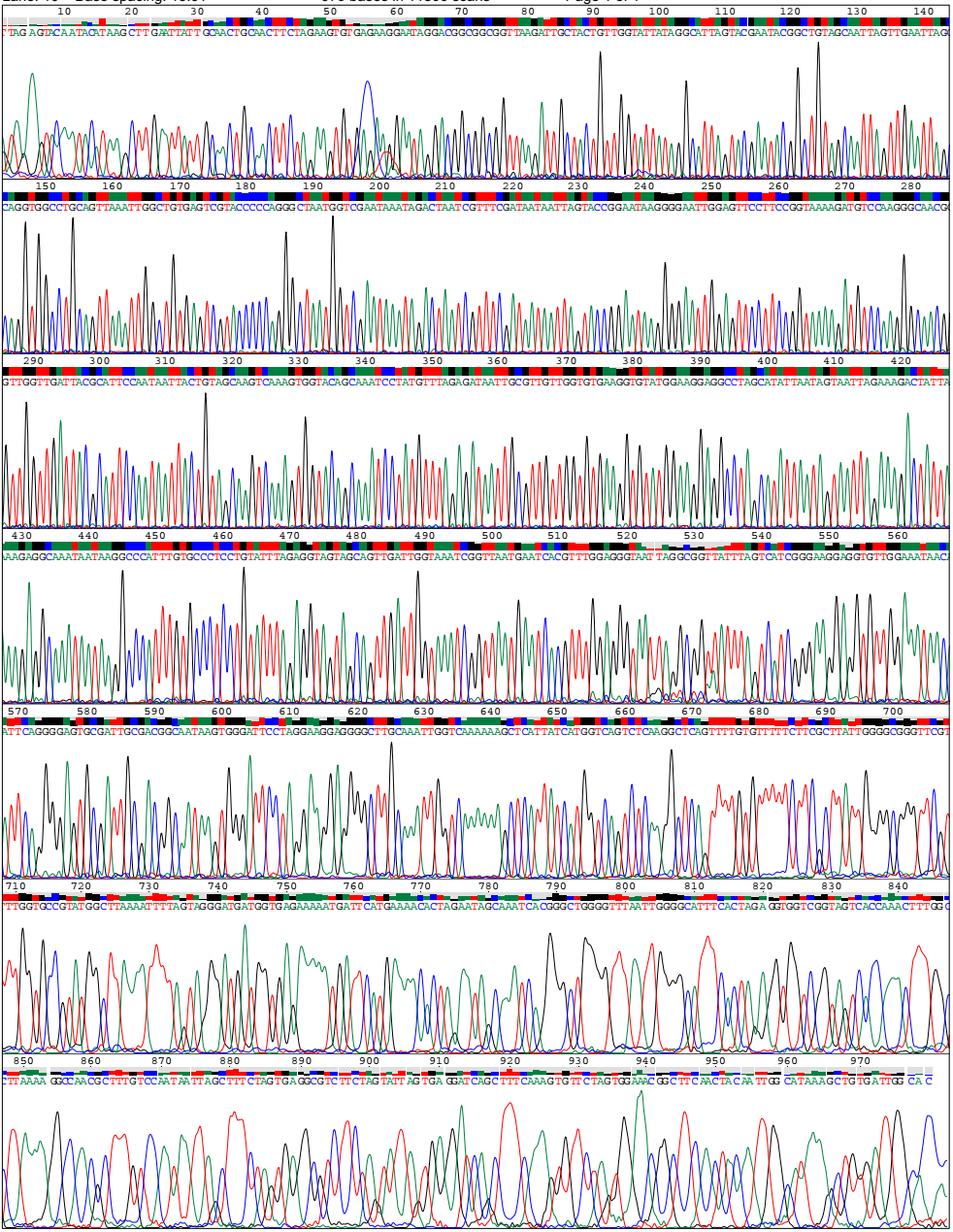


1. Fragment 7 was sequenced using primer pair 7 as follow:

Fragment 7-anterior sequence (forward sequencing)


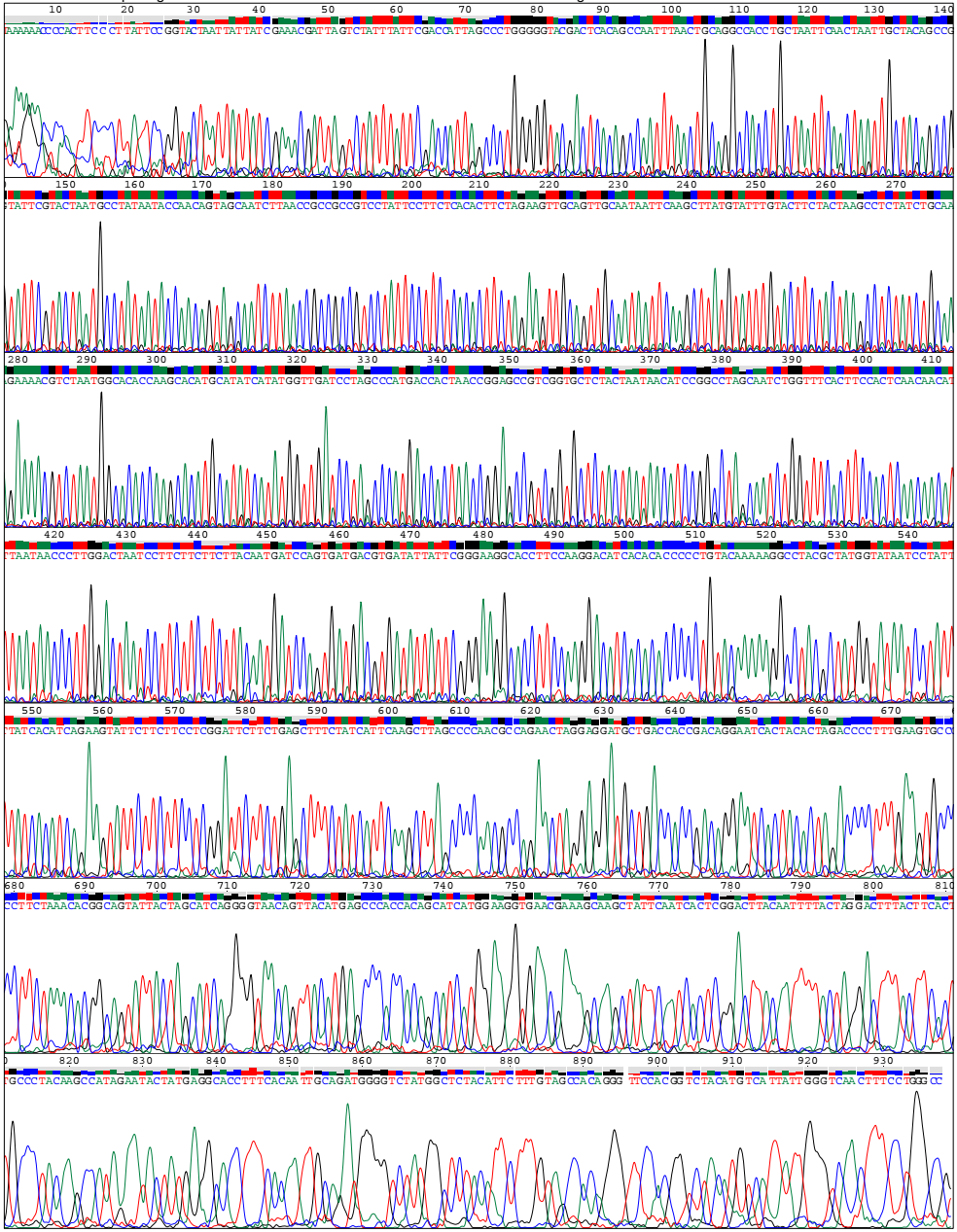


Fragment 7-posterior sequence (reverse sequencing)


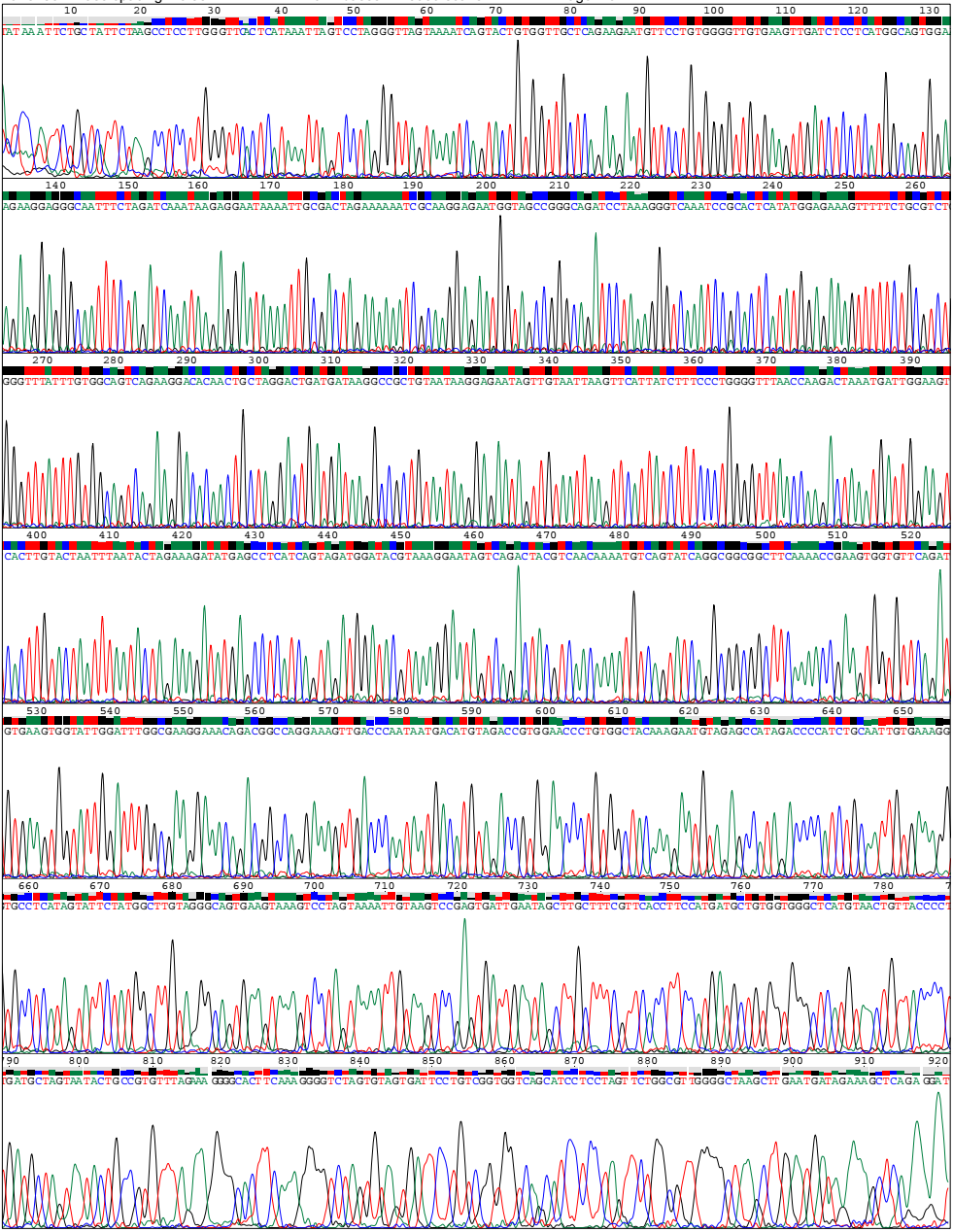


1. Fragment 8 was sequenced only using the reverse primer of primer pair 8. (The obtained sequence of fragment 8 could overlap with Fragment 7-posterior sequence and Fragment 9-anterior sequence)

Fragment 8 (reverse sequencing)


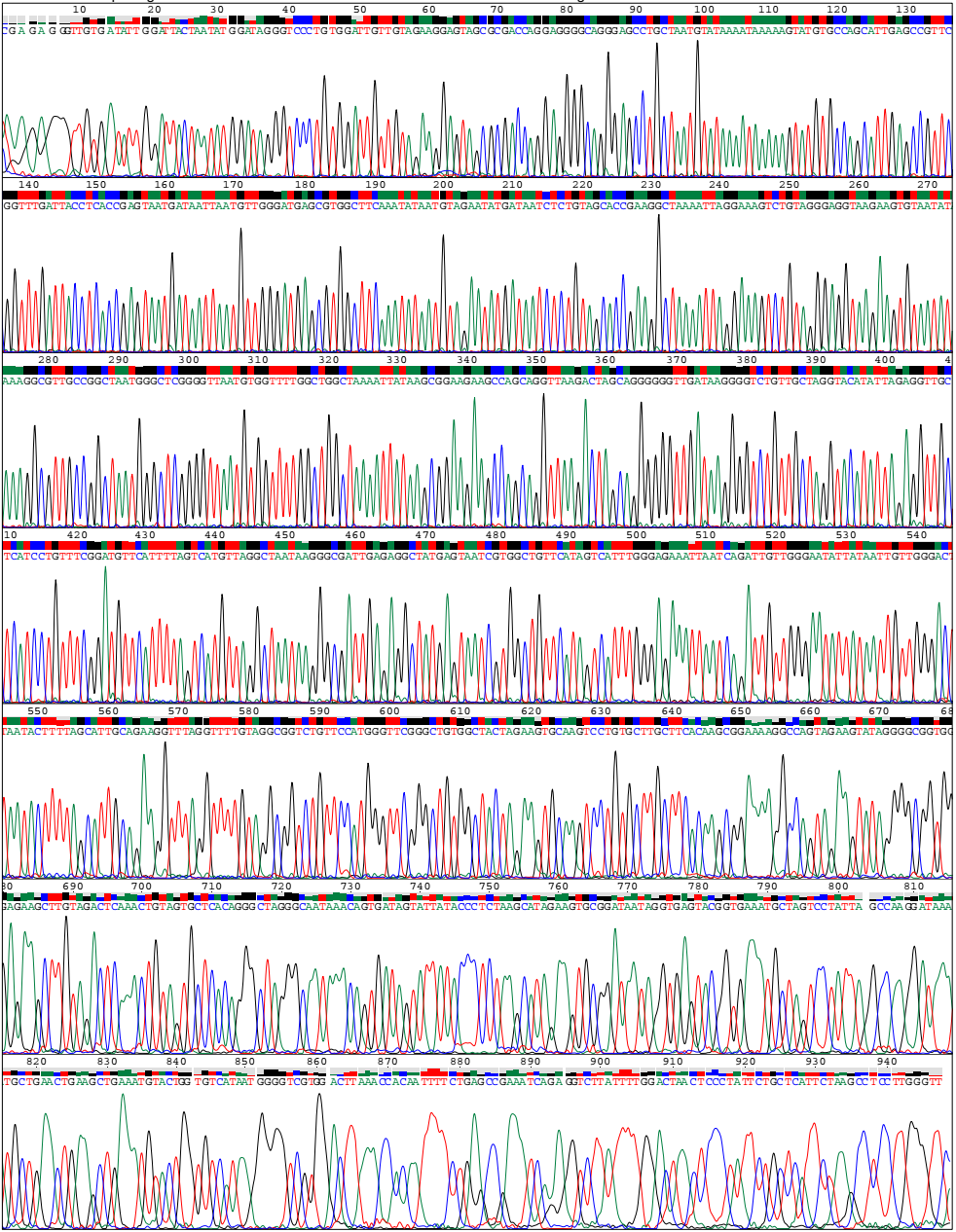


1. Fragment 9 was sequenced using primer pair 9 as follow:

Fragment 9-anterior sequence (forward sequencing)


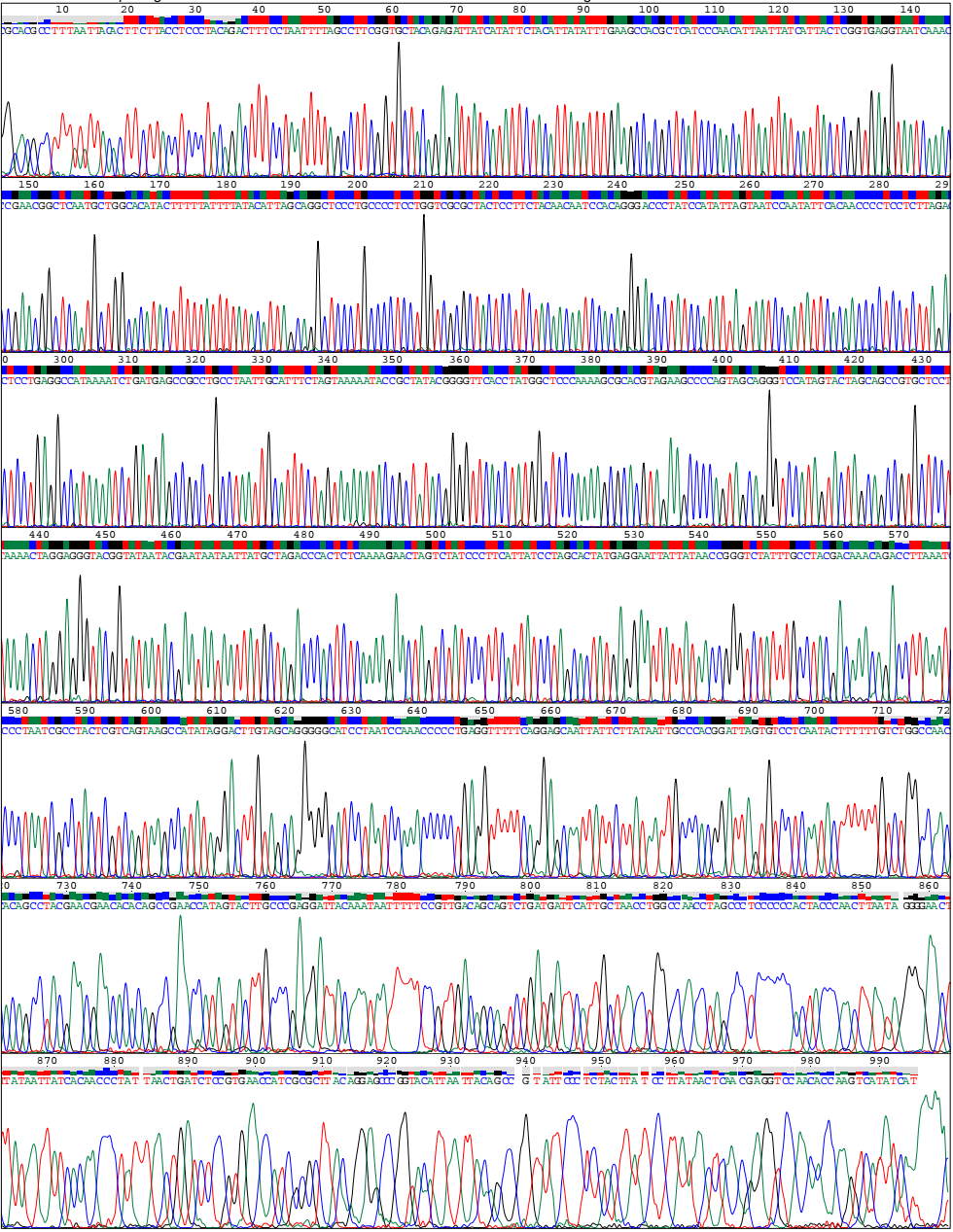


Fragment 9-posterior sequence (reverse sequencing)


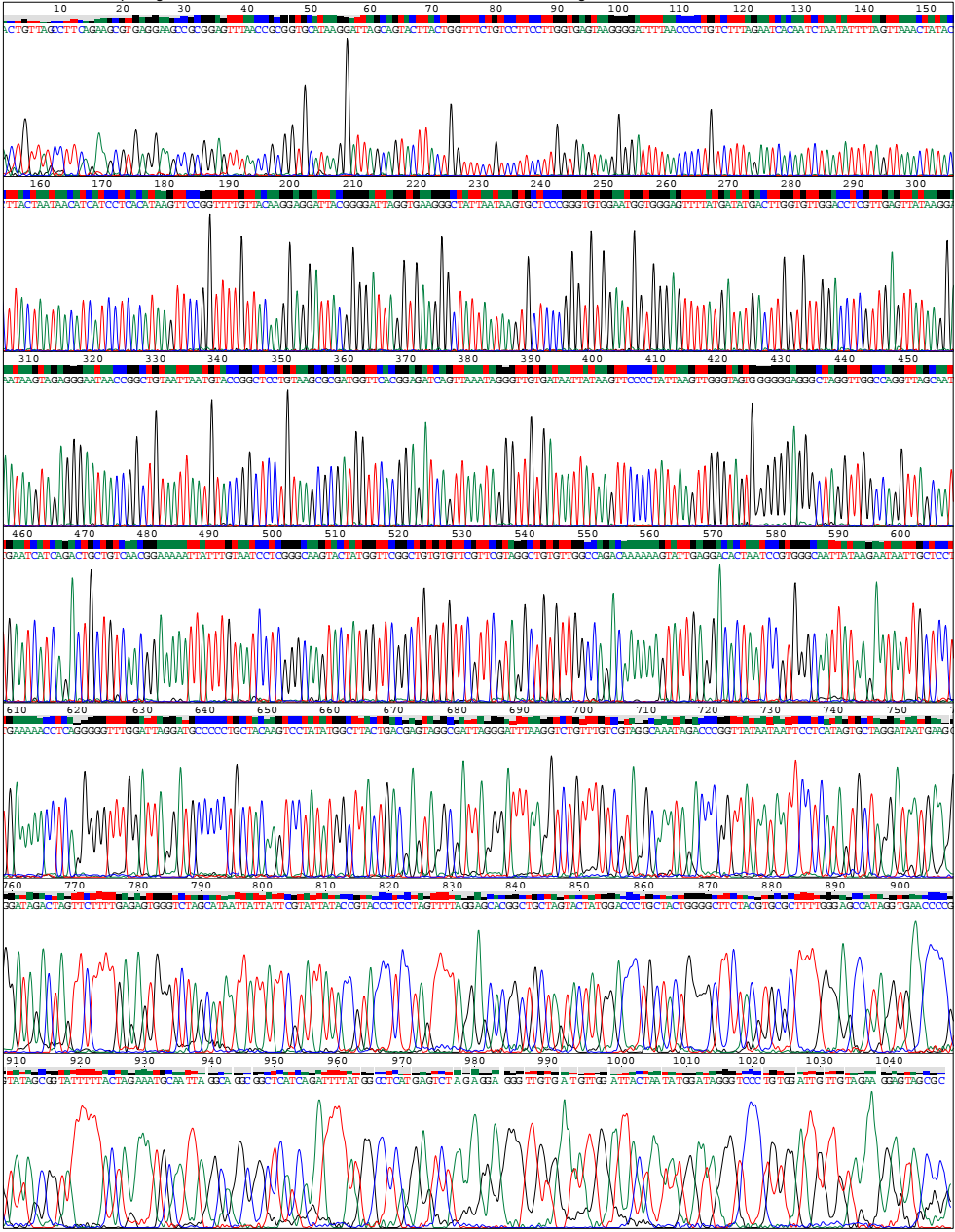


1. Fragment 10 was sequenced using primer pair 10 as follow:

Fragment 10-anterior sequence (forward sequencing)


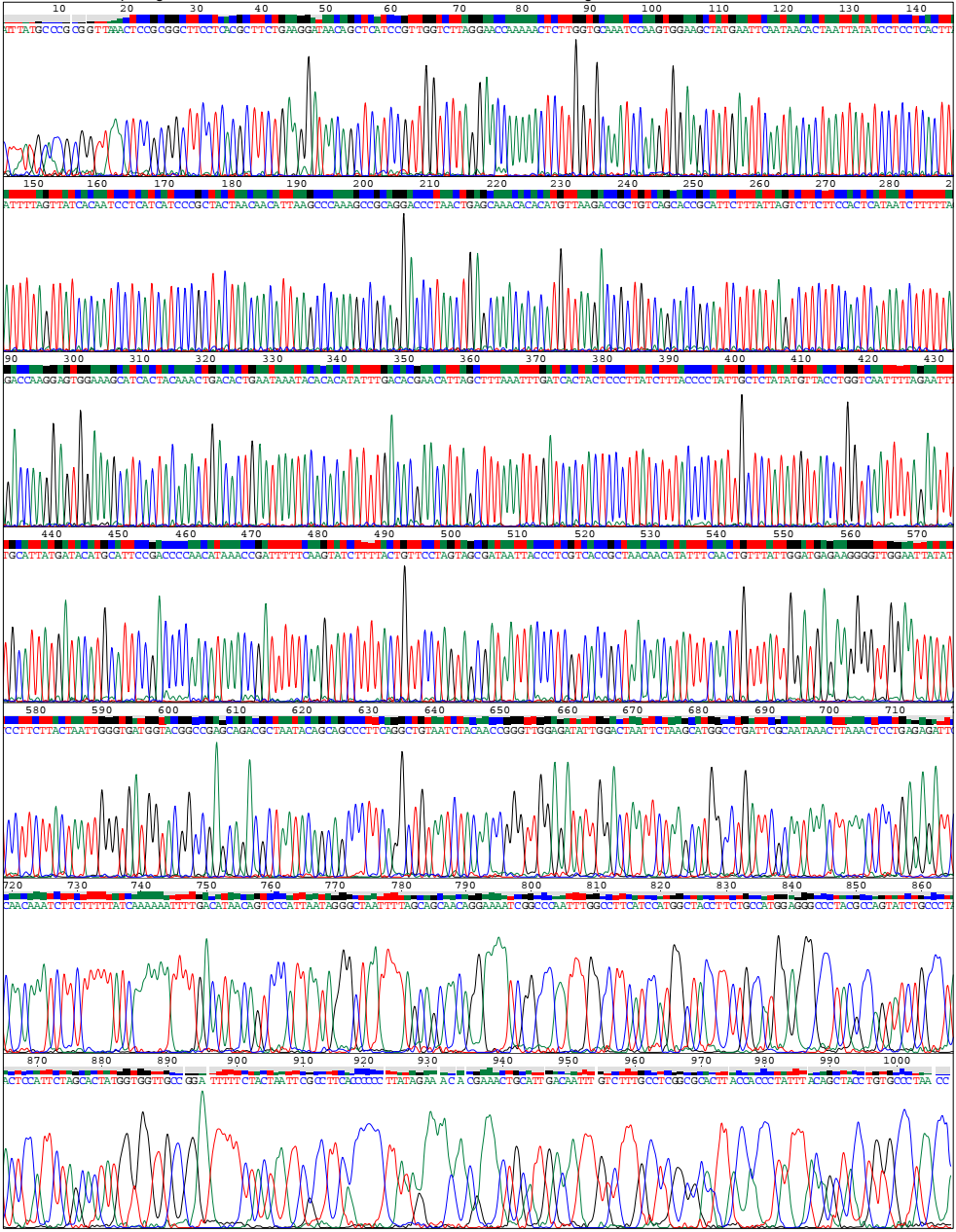


Fragment 10-posterior sequence (reverse sequencing)


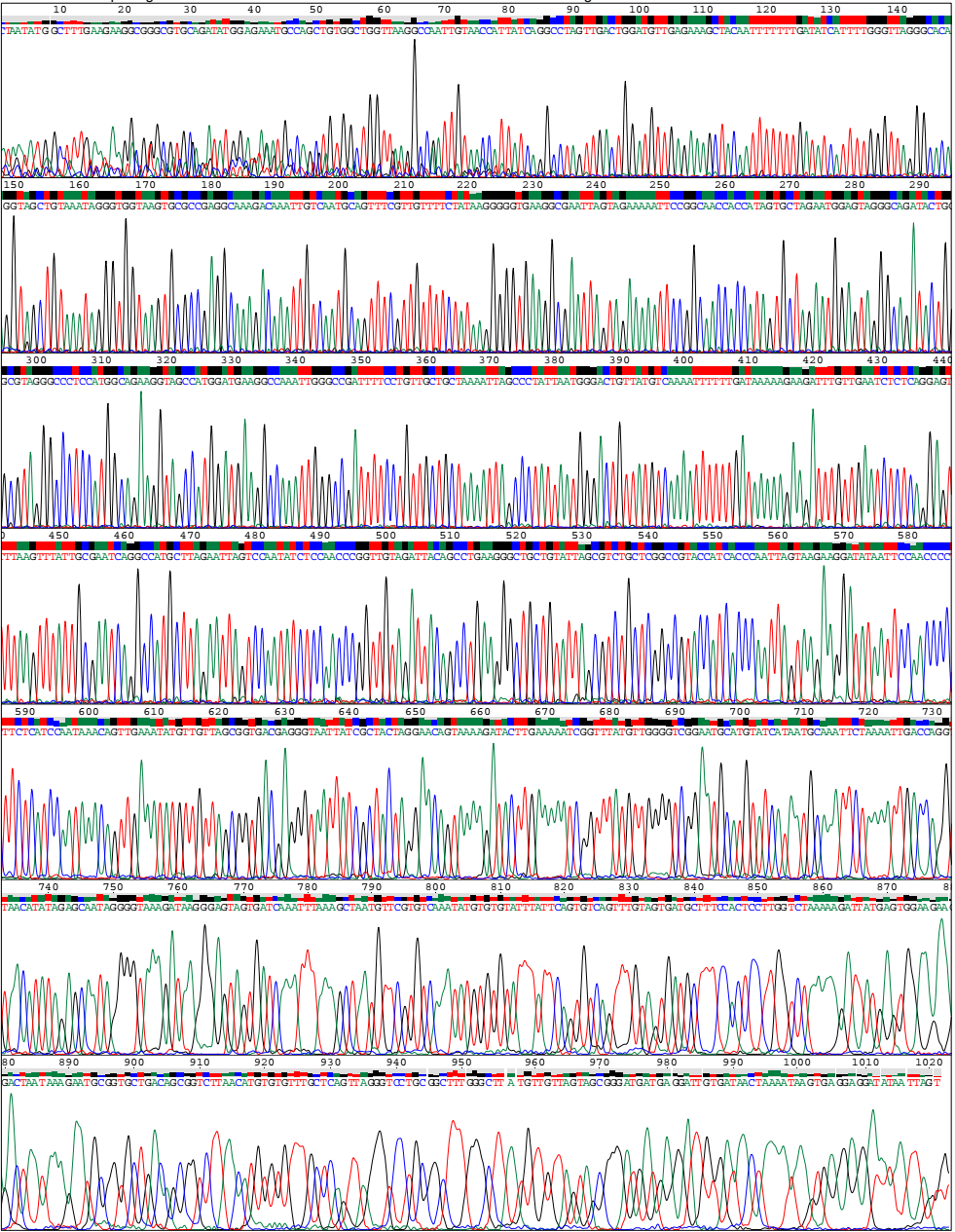


1. Fragment 11 was sequenced using primer pair 11 as follow:

Fragment 11-anterior sequence (forward sequencing)


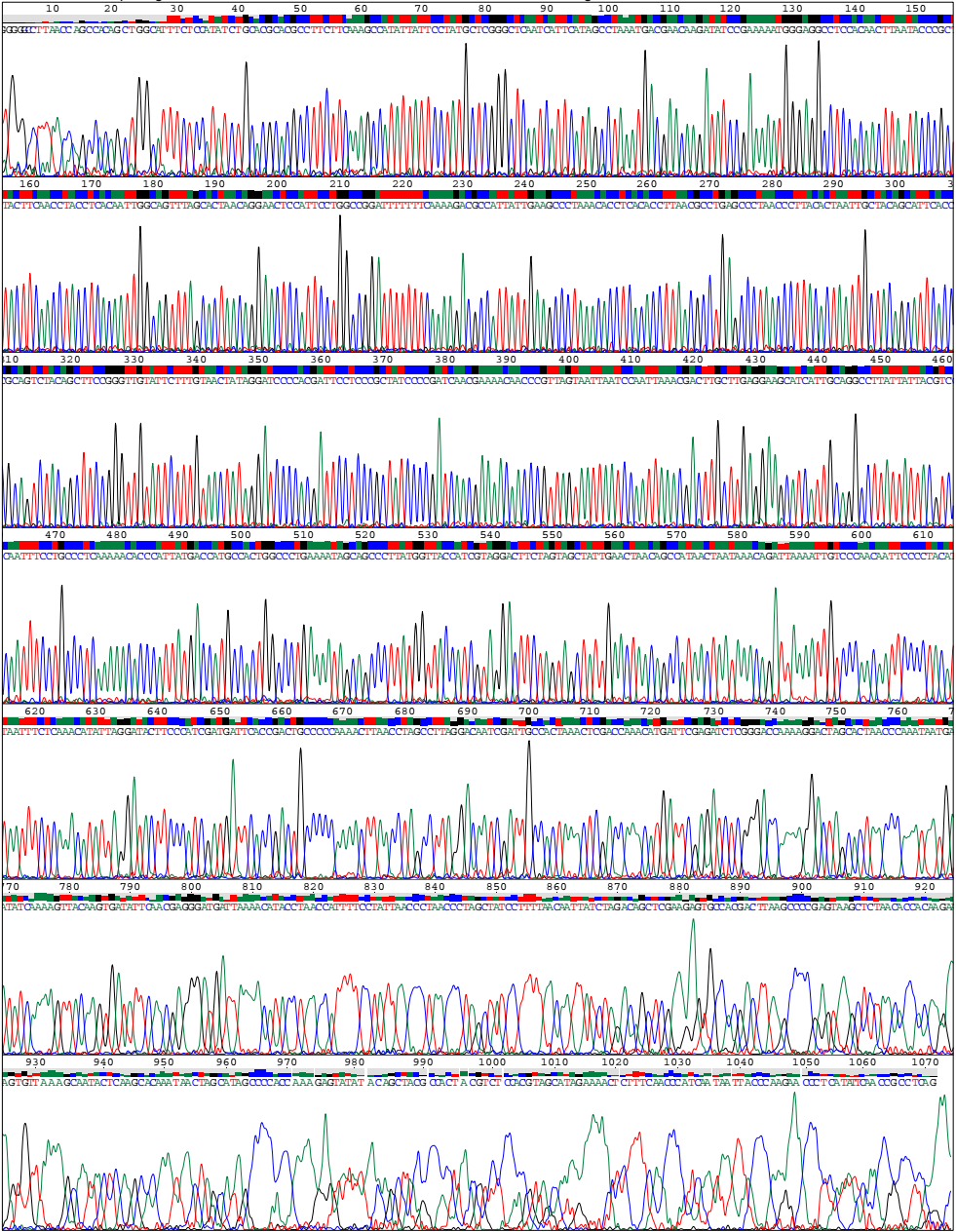


Fragment 11-posterior sequence (reverse sequencing)


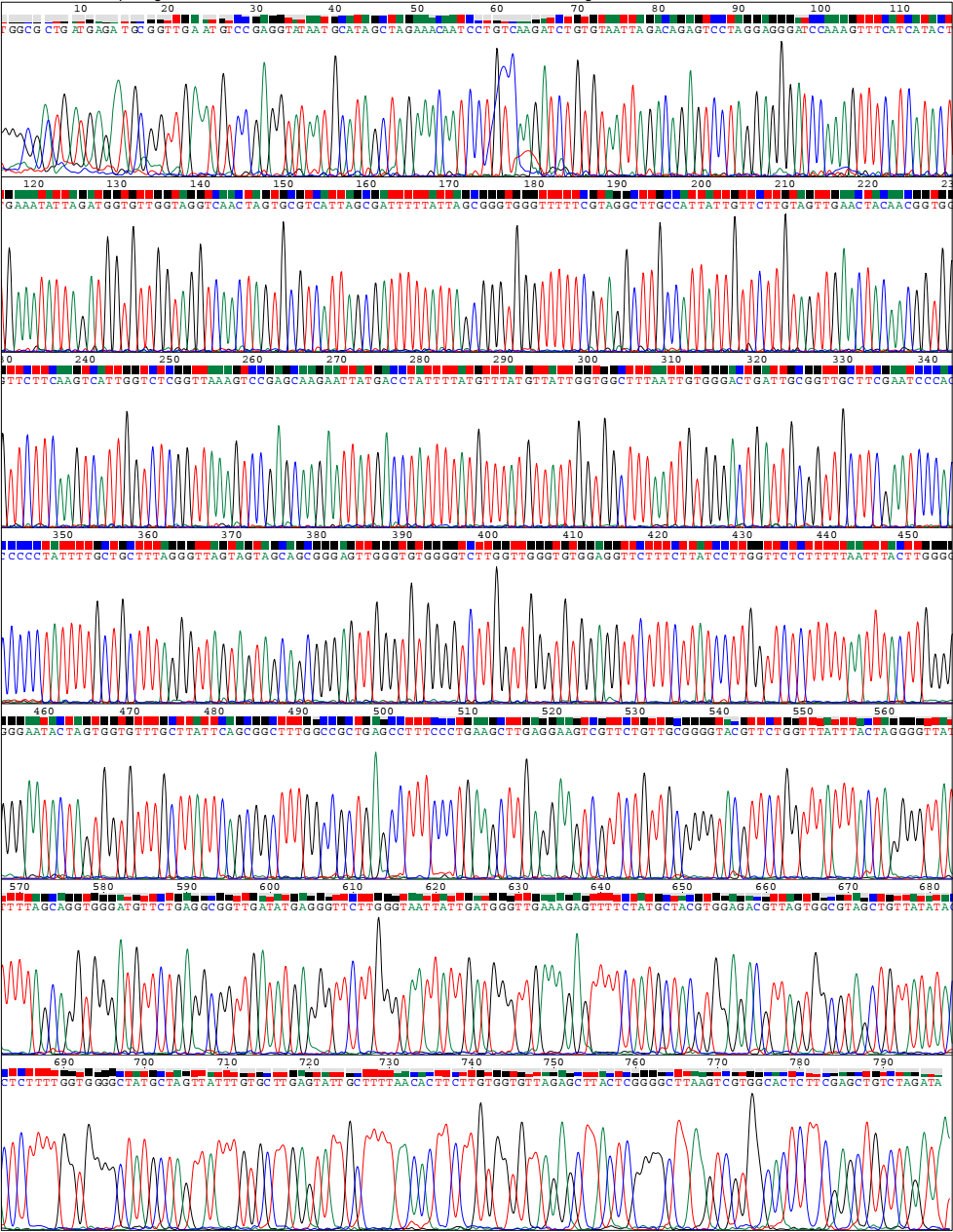


1. Fragment 12 was sequenced using primer pair 12 as follow:

Fragment 12-anterior sequence (forward sequencing)


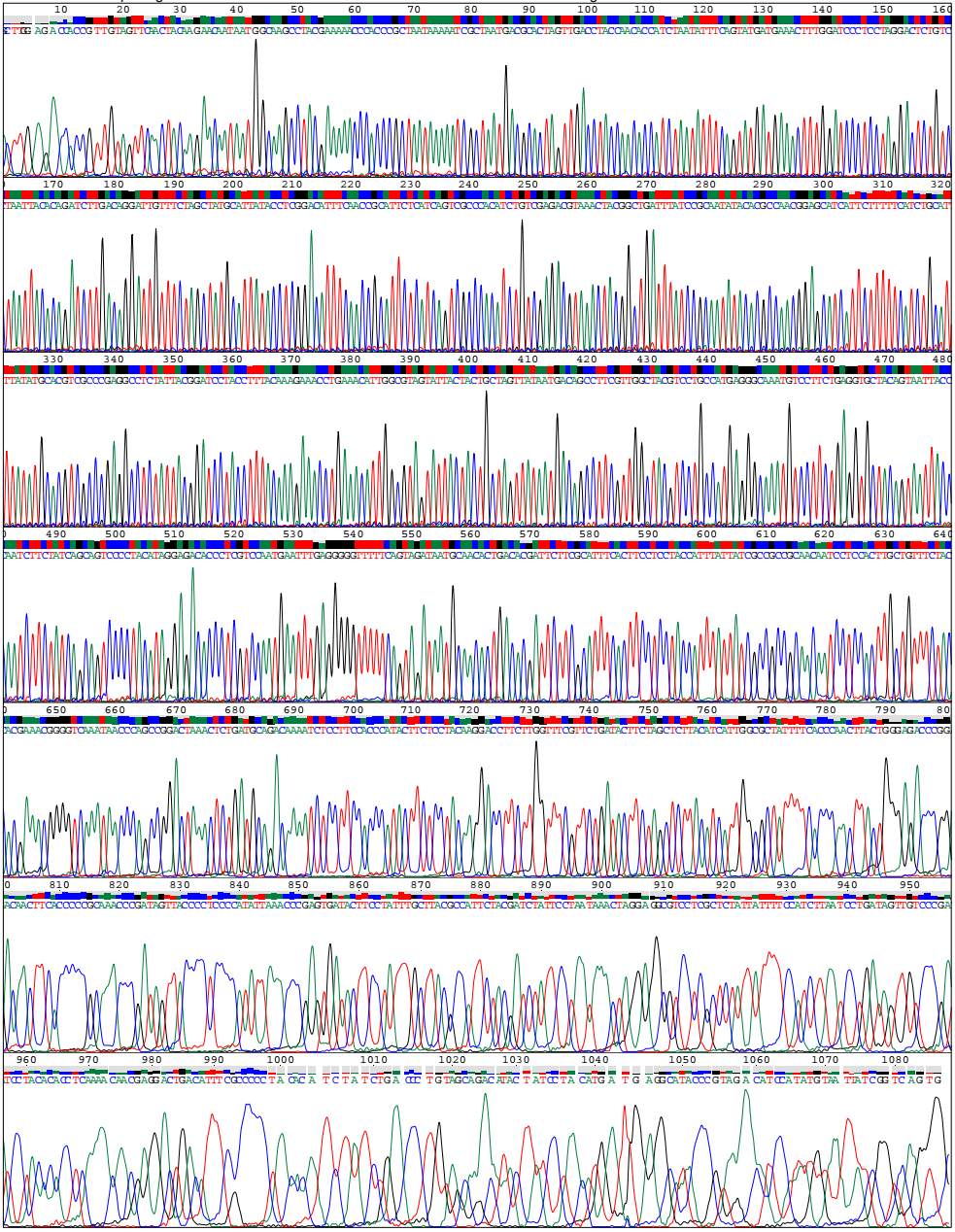


Fragment 12-posterior sequence (reverse sequencing)


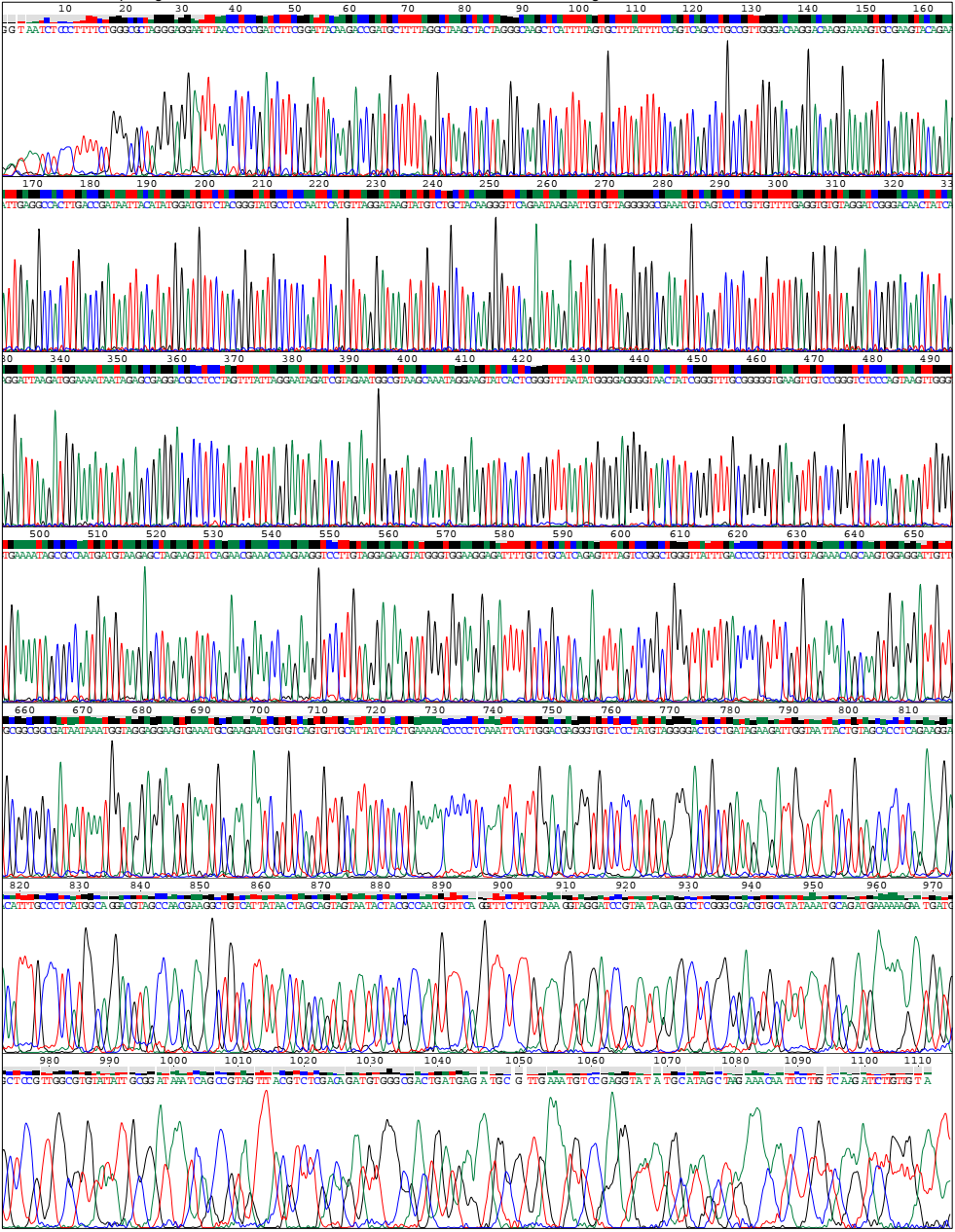


1. Fragment 13 was sequenced using primer pair 13 as follow:

Fragment 13-anterior sequence (forward sequencing)


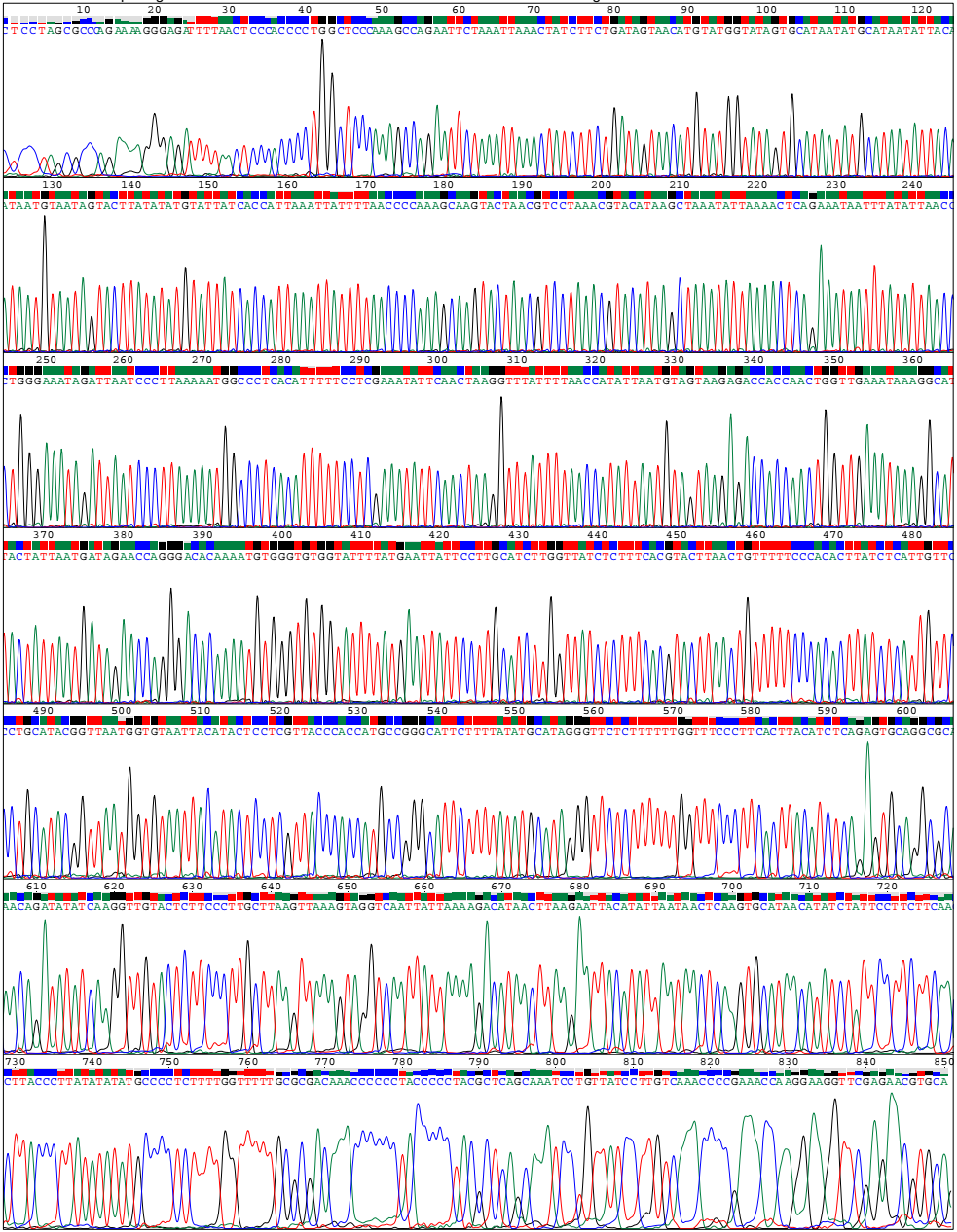


Fragment 13-posterior sequence (reverse sequencing)


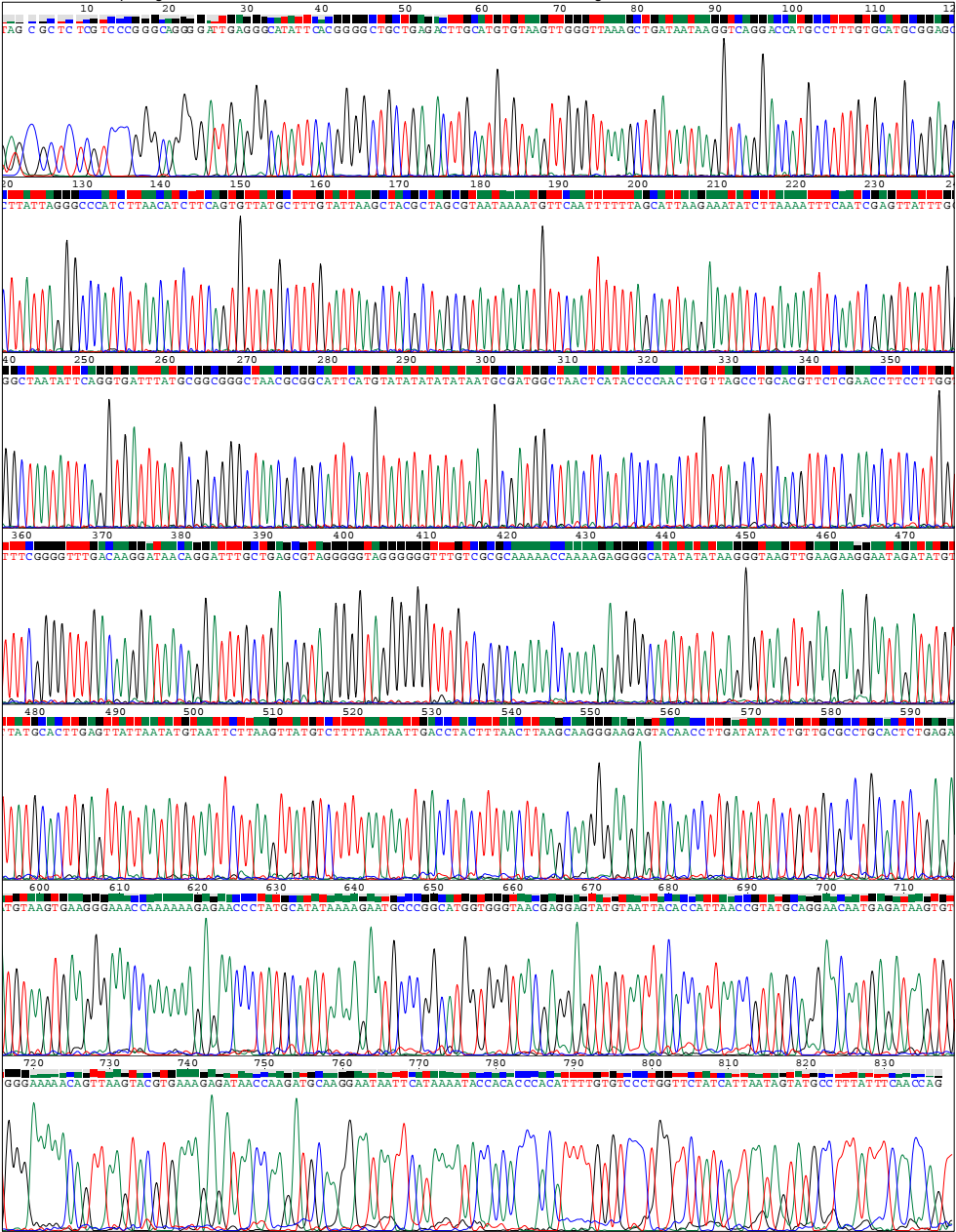

Supplement: Supplemental Material [file TMDN_A_2131370_SM7407.docx]
