# Supplementary material for: The complete mitochondrial genome of Mesogobio lachneri (Cypriniformes: Gobionidae) from Northeast Asia
Source: Mitochondrial DNA B Resour. 2022 Oct 19;7(10):1810–3. doi: 10.1080/23802359.2022.2131370 (PMC9586598; doi:10.1080/23802359.2022.2131370)
Supplement: Supplemental Material [file TMDN_A_2131370_SM7406.docx]

**Supplementary Material—Appendix I**

**The complete mitochondrial genome of *Mesogobio lachneri* (Cypriniformes: Gobionidae) from Northeast Asia**

Wei Tian, Xiaomin Ni and Cuizhang Fu*


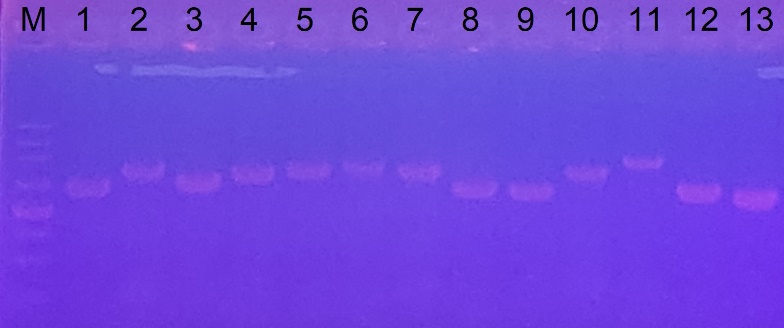


Figure S1 PCR gel image for amplification of mitochondrial genome for *Mesogobio lachneri*. M indicated 250 bp DNA Marker, and lane 1–13 indicated fragment 1–13 amplified using 13 primer pairs. Photographs by Wei Tian at November 15th, 2021.

Table S1 Thirteen primer pairs used in this study

| Primer pair | Forward primer | Reverse primer | Amplification length | Source |
| --- | --- | --- | --- | --- |
| 1 | Gob12sF 5'-AAGGCATGGTCCYGACCTTA-3' | Gob16sR: 5'-TTCGGTAGGTCTRTCACTTC-3' | 1403 bp | Chai and Fu, 2020 |
| 2 | Gob16sF: 5'-ACCTTGTACCTTTTGCATC-3 | GobLeuR: 5'-GGGAAGAGGAYTTGAACC-3' | 1560 bp | Chai and Fu, 2020 |
| 3 | GobND1F: 5'-GCAGCCGCTATTAAGGGTT-3' | GobND1R: 5'-GGRTTCATTGATGGAGGA-3' | 1501bp | Chai and Fu, 2020 |
| 4 | GobIleF: 5'-GCCCAAGGACCACTTTGATAG-3' | GobCOIR: 5'-CCAAATACRAGATARAGGT-3' | 1692 bp | Chai and Fu, 2020 |
| 5 | GobAsnF: 5'-AGCGAGCATCCATCTACTT-3' | GobSerR: 5'-GGTYATGTGACTGGCTTGA-3' | 1795 bp | Chai and Fu, 2020 |
| 6 | GobCOIF: 5'-TGAGAAGCCTTYGCCGCYAAACG'-3' | GobATP6R: 5'-AGGAATACYATYAGGGAGGC-3' | 1759 bp | Chai and Fu, 2020 |
| 7 | GobATP6F: 5'-CCTTGAGAYTGACCATGAT-3' | GobArgR: 5'-CTGAGYCGAAATCAGAGG-3' | 1593 bp | Chai and Fu, 2020 |
| 8 | GobCOIIIF: 50-TGATGAGGCTCATATCTTTCTA-30 | MeLND4R: 5'-TAAAATCTGRTGGGCCGG-3' | 1355 bp | Chai and Fu, 2020; This study |
| 9 | MeLND4F: 5'-TAGCCAGCCAAAAYCACAT-3' | GobLeuR: 5'-TGGAYTTGCACCAAGAGT-3' | 1350 bp | This study; Chai and Fu, 2020 |
| 10 | GobSerF: 5'-ACTYACCRAGGAAGGACA-3' | MeLND5R: 5'-AAACGRCTTGCCTGRGGAAG-3' | 1553 bp | Chai and Fu, 2020; This study |
| 11 | GobND5F: 5'-ATTGARGCCCTAAACACCTC-3' | CGobCytbR: 5'-AA GTGGAAKGCGAARAATCG-3' | 1972 bp | Chai and Fu, 2020 |
| 12 | GobND6F: 5'-AAAATAGGTCATAATTCTTGCTCGG-3' | GobProR: 5'-GTTTAATTTAGAATTCTGGCTTTGG-3' | 1385 bp | Chai and Fu, 2020 |
| 13 | GobDloopF: 5'-AAAGCATCGGTCTTGTAATC-3' | GobDloopR: 5'-CTTGGCTAGGCGTCTTGG-3' | 1275 bp | Chai and Fu, 2020 |
